# A machine learning route between band mapping and band structure

**DOI:** 10.1038/s43588-022-00382-2

**Published:** 2022-12-30

**Authors:** R. Patrick Xian, Vincent Stimper, Marios Zacharias, Maciej Dendzik, Shuo Dong, Samuel Beaulieu, Bernhard Schölkopf, Martin Wolf, Laurenz Rettig, Christian Carbogno, Stefan Bauer, Ralph Ernstorfer

**Affiliations:** 1 Fritz Haber Institute of the Max Planck Society, Berlin, Germany; 2 Department of Empirical Inference, Max Planck Institute for Intelligent Systems, Tübingen, Germany; 3 Present Address: Department of Mechanical Engineering, University College London, London, UK; 4 Present Address: Université de Rennes, INSA Rennes, CNRS, Institut FOTON, Rennes, France; 5 Present Address: Department of Applied Physics, KTH Royal Institute of Technology, Stockholm, Sweden; 6 Present Address: Université de Bordeaux-CNRS-CEA, CELIA, Talence, France; 7 Present Address: Division of Decision and Control Systems, KTH Royal Institute of Technology, Stockholm, Sweden

**Keywords:** Electronic properties and materials, Software, Computational methods

## Abstract

The electronic band structure and crystal structure are the two complementary identifiers of solid-state materials. Although convenient instruments and reconstruction algorithms have made large, empirical, crystal structure databases possible, extracting the quasiparticle dispersion (closely related to band structure) from photoemission band mapping data is currently limited by the available computational methods. To cope with the growing size and scale of photoemission data, here we develop a pipeline including probabilistic machine learning and the associated data processing, optimization and evaluation methods for band-structure reconstruction, leveraging theoretical calculations. The pipeline reconstructs all 14 valence bands of a semiconductor and shows excellent performance on benchmarks and other materials datasets. The reconstruction uncovers previously inaccessible momentum-space structural information on both global and local scales, while realizing a path towards integration with materials science databases. Our approach illustrates the potential of combining machine learning and domain knowledge for scalable feature extraction in multidimensional data.

## Abstract (web summary)

A computational workflow centered on probabilistic machine learning is developed to reconstruct the energy dispersion from photoemission band-mapping data. The workflow uncovers previously inaccessible momentum-space structural information at scale.

## Main

**Fig. 1:**
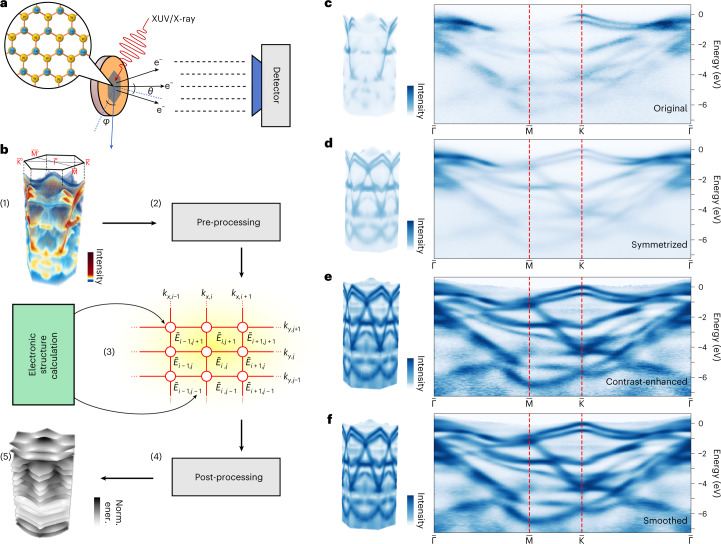
From band mapping to BS. **a**, Schematic of a photoemission band-mapping experiment. The electrons from a crystalline sample’s surface are liberated by extreme-ultraviolet (XUV) or X-ray pulses and collected by a detector through either angular scanning or time-of-flight detection schemes. **b**, Overview of the computational framework for reconstruction of the photoemission (or quasiparticle) BS: (1) the volumetric data obtained from a band-mapping experiment (2) go through pre-processing steps, then are (3) fed into the probabilistic machine-learning algorithm along with electronic structure calculations as initialization of the optimization. The reconstruction algorithm for volumetric band-mapping data is represented as a 2D probabilistic graphical model with the band energies as nodes, leading to tens of thousands of nodes in practice. (4) The outcome of the reconstruction is post-processed (for example, symmetrization) to (5) yield the dispersion surfaces (energy bands) of the photoemission BS ordered by band indices. **c**–**f**, Effects of the intensity transforms in data pre-processing viewed in both 3D and along the high-symmetry line of the projected Brillouin zone (hexagonal as in **b**(1)), starting from the original data (**c**) through intensity symmetrization (**d**), contrast enhancement^29^ (**e**) and Gaussian smoothing of intensities (**f**). The intensity data in **c**–**f** are normalized individually for visual comparison. Source data

Modeling and characterization of the electronic band structure (BS) of a material play essential roles in materials design^1^ and device simulation^2^. The BS exists in momentum space, *Ω*(*k*_*x*_, *k*_*y*_, *k*_*z*_, *E*), and imprints the multidimensional and multivalued functional relations between the energy (*E*) and momenta (*k*_*x*_, *k*_*y*_, *k*_*z*_) of periodically confined electrons^3^. Photoemission band mapping^4^ (Fig. 1a) using momentum- and energy-resolved photoemission spectroscopy (PES), including angle-resolved PES (ARPES)^5,6^ and multidimensional PES^7,8^, measures the BS as an intensity-valued multivariate probability distribution directly in *Ω*. The proliferation of band-mapping datasets and their public availability brought about by recent hardware upgrades^7–10^ have ushered in possibilities regarding the comprehensive benchmarking of theories and experiments, which is especially challenging for multiband materials with complex band dispersions^11–13^. The available methods for interpreting photoemission spectra fall into two categories: physics-based methods, which require least-squares fitting of one-dimensional lineshapes, named energy or momentum distribution curves (EDCs or MDCs), and analytical models^5,14,15^. Although physics-informed data models guarantee high accuracy and interpretability, upscaling the pointwise fitting (or estimation) to large, densely sampled regions in momentum space (for example, including 10^4^ or more momentum locations) presents challenges due to the limited numerical stability and efficiency. Therefore, their use is limited to selected momentum locations determined heuristically from physical knowledge of the materials and experimental settings. Image-processing-based methods apply data transformations to improve the visibility of dispersive features^16–19^. They are more computationally efficient and can operate on entire datasets, yet offer only visual enhancement of the underlying band dispersion. They do not allow reconstruction and are therefore insufficient for truly quantitative benchmarking or archiving.

A method balancing the two approaches will extract the band dispersion with sufficiently high accuracy and be scalable to multidimensional datasets, therefore providing the basis for distilling structural information from complex band-mapping data and for building efficient tools for annotating and understanding spectra. In this regard we propose a computational framework (Fig. 1b) for global reconstruction of the photoemission (or quasiparticle) BS as a set of energy (or electronic) bands, formed by energy values (that is, band loci) connected along momentum coordinates. This local connectedness assumption is more valid than using local maxima of photoemission intensities, because local maxima are not always good indicators of band loci^20^. We exploit the connection between theory and experiment in our framework, based on a probabilistic machine-learning^21,22^ model, to approximate the intensity data from band-mapping experiments. The gist of the model is rooted in Bayes rule:

1 $${p}({{{{X}}}}| {{{\mathcal{D}}}})\propto {p}({{{\mathcal{D}}}}| {{{{X}}}}){p}({{{{X}}}}),$$

where *X* are the random variables to be inferred and the data ${{{\mathcal{D}}}}$ are mapped directly onto unknowns and experimental observables. We assign the energy values of the photoemission BS as the model’s variables to extract from data, and a nearest-neighbor (NN) Gaussian distribution as the prior, *p*(*X*), to describe the proximity of energy values at nearby momenta. The EDC at every momentum grid point relates to the likelihood, ${p}({{{\mathcal{D}}}}| {{{{X}}}})$, when we interpret the photoemission intensity probabilistically. The optimum is obtained via maximum a posteriori (MAP) estimation in probabilistic inference^21^ (Methods and Supplementary Fig. 2). Given the form of the NN prior, the posterior, ${p}({{{{X}}}}| {{{\mathcal{D}}}})$, in the current setting forms a Markov random field (MRF)^21,23,24^, which encapsulates the energy-band continuity assumption and the measured intensity distribution of photoemission in a probabilistic graphical model. In one benefit, the probabilistic formulation can incorporate imperfect physical knowledge algebraically in the model or numerically as the initialization (that is, warm start; Methods) of the MAP estimation, without requiring the de facto ground truth and training as in supervised machine learning^25^. In another benefit, the graphical model representation allows convenient optimization and extension to other dimensions (Supplementary Fig. 1 and Supplementary Section 1).

To demonstrate the effectiveness of the method, we first reconstructed the entire 3D dispersion surface, *E*(*k*_*x*_, *k*_*y*_), of all 14 valence bands within the projected first Brillouin zone (in (*k*_*x*_, *k*_*y*_, *E*) coordinates) of the semiconductor tungsten diselenide (WSe_2_), spanning ~7 eV in energy and ~3 Å^−1^ along each momentum direction. We also adapted the informatics tools to BS data to sample and compare the reconstructed and theoretical BSs globally. The accuracy of the reconstruction was validated using synthetic data and the extracted local structural parameters along with pointwise fitting. The available data and BS informatics enable a detailed comparison of band dispersion at a resolution of <0.02 Å^−1^. We performed various tests and benchmarking on datasets of other materials and simulated data, where ground truth is available to evaluate the accuracy and computational efficiency.

## Results

### BS reconstruction and digitization

Our main example is the 2D layered semiconductor WSe_2_, with its hexagonal lattice and bilayer stacking periodicity (denoted 2*H*-WSe_2_), as a model system for band-mapping experiments^11,26,27^. Earlier valence-band mapping and reconstruction in ARPES experiments on WSe_2_ demonstrated a high degree of similarity between theory and experiments^11,26,27^, but a quantitative assessment within the entire (projected) Brillouin zone is still lacking. The valence BS of 2*H*-WSe_2_ contains 14 strongly dispersive energy bands, formed by a mixture of the 5*d*^4^ and 6*s*^2^ orbitals of the W atoms and the 4*p*^4^ orbitals of the Se atoms, in its hexagonal unit cell. The strong spin–orbit coupling (SOC) due to these heavy elements produces large momentum- and spin-dependent energy splitting and modifications to the BS^11,28^.

We use a 2D MRF to model the loci of an energy band within the intensity-valued 3D band-mapping data, regarded as a collection of momentum-ordered EDCs. This is graphically represented by a rectangular grid overlaid on the momentum axes with indices (*i*, *j*) (where *i*, *j* are non-negative integers), as shown in step (3) of Fig. 1b. The undetermined band energy of the EDC at (*i*, *j*), with the associated momentum coordinates (*k*_*x*, *i*_, *k*_*y*, *j*_), is considered a random variable, ${\tilde{E}}_{i,\,j}$, of the MRF. Together, the probabilistic model is characterized by a joint distribution, expressed as the product of the likelihood and the Gaussian prior in equation (1). To maintain its simplicity, we do not explicitly account for the intensity modulations of various origins (such as imbalanced transition matrix elements^20^) in the original band-mapping data, which cannot be remediated by upgrading the photon source or detector. Instead, we pre-process the data to minimize their effects on the reconstruction (Fig. 1c–f). The pre-processing steps include (1) intensity symmetrization and (2) contrast enhancement^29^, followed by (3) Gaussian smoothing (Methods), after which the continuity of band-like features is restored. The EDCs from the pre-processed data, ${\tilde{I}}$, are used effectively as the likelihood to calculate the MRF joint distribution:

2 $${p}(\{{\tilde{E}}_{i,\,j}\})={\frac{1}{Z}\mathop{\prod}\limits_{ij}\tilde{I}({k}_{x,\,i},\,{k}_{y,\,j},\,{\tilde{E}}_{i,\,j})\cdot \mathop{\prod}\limits_{(i,\,j)(l,\,m)| {{{\rm{NN}}}}}\exp \left[-\frac{{({\tilde{E}}_{i,\,j}-{\tilde{E}}_{l,\,m})}^{2}}{2{\eta }^{2}}\right]}.$$

Here, *Z* is a normalization constant, *η* is a hyperparameter defining the width of the Gaussian prior, ∏_*ij*_ denotes the product over all discrete momentum values sampled in the experiment, and ∏_(*i*, *j*)(*l*, *m*)∣NN_ is the product over all NN terms. A detailed derivation of equation (2) is given in Supplementary Section 1. Reconstruction of the photoemission BS is carried out sequentially for all bands and relies on local optimization of the MRF’s variables, ${\{{\tilde{E}}_{i,\,j}\}}$.

To optimize over large graphical models, we adopt multiple parallelization schemes to achieve efficient operations on scalable computing hardware. A single band reconstruction involving optimization over 10^4^ random variables is achieved within seconds and hyperparameter tuning within tens of minutes (Methods and Supplementary Figs. 3 and 4). In comparison, pointwise fitting often requires individual hand-tuning and is therefore difficult to scale up to whole bands within a meaningful timeframe. To correctly resolve band crossings and nearly degenerate energies, we inject relevant physical knowledge into the optimization by using density functional theory (DFT) BS calculations with semi-local approximation^30^ as a starting point for the reconstruction. The calculation qualitatively involves physical symmetry information for WSe_2_, albeit not quantitatively reproducing the experimental quasiparticle BSs at all momentum coordinates. As shown with four DFT calculations with different exchange-correlation functionals^30^ to initiate the reconstruction for WSe_2_ and in various cases using synthetic data with known ground truths (Methods, Supplementary Table 3 and Supplementary Figs. 4–8), the reconstruction algorithm is not particularly sensitive to the initialization as long as the information about band crossings is present. The current framework can also support initialization from more advanced electronic-structure methods, such as GW^31^ or those including electronic self-energies renormalized by electron–phonon coupling^32^, where semi-local approximation yields not only quantitatively, but also qualitatively wrong quasiparticle BSs compared with the experiment. However, a systematic benchmarking of theory and experiment goes beyond the scope of this work.

**Fig. 2:**
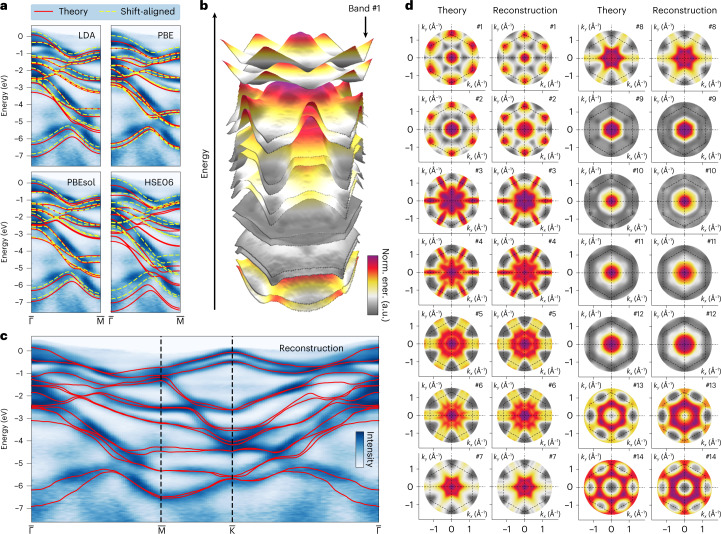
Band reconstruction from WSe_2_ photoemission data. **a**, Comparison between the pre-processed WSe_2_ valence-band photoemission data along the ${\overline{{{\Gamma }}}}$–${\overline{{{{\rm{M}}}}}}$ direction, the DFT BS calculated with different exchange-correlation functionals (solid red lines), and their final positions after band-wise rigid-shift alignment (dashed yellow lines) as part of hyperparameter tuning. The energy zero of each DFT calculation is set at the ${\overline{{{{\rm{K}}}}}}$ point (not shown). **b**, Exploded view (with enlarged spacing between bands for better visibility) of the reconstructed energy bands of WSe_2_. **c**, Overlay of the reconstructed band dispersion (red lines) on the pre-processed photoemission band-mapping data, cut along the high-symmetry line of the hexagonal Brillouin zone of WSe_2_. **d**, Band-wise comparison between the LDA-level DFT (LDA-DFT) calculation used to initialize the optimization and the 14 reconstructed valence bands of WSe_2_ (symmetrized in post-processing). The dashed hexagons trace out the boundaries of the first Brillouin zone. The band indices on the upper right corners in **d** follow the ordering of the electronic orbitals in this material, obtained from LDA-DFT. **b** and **d** are paired plots (Methods) that share the same color bar, which shows the per-band normalized energy in arbitrary units (a.u.). Source data

**Fig. 3:**
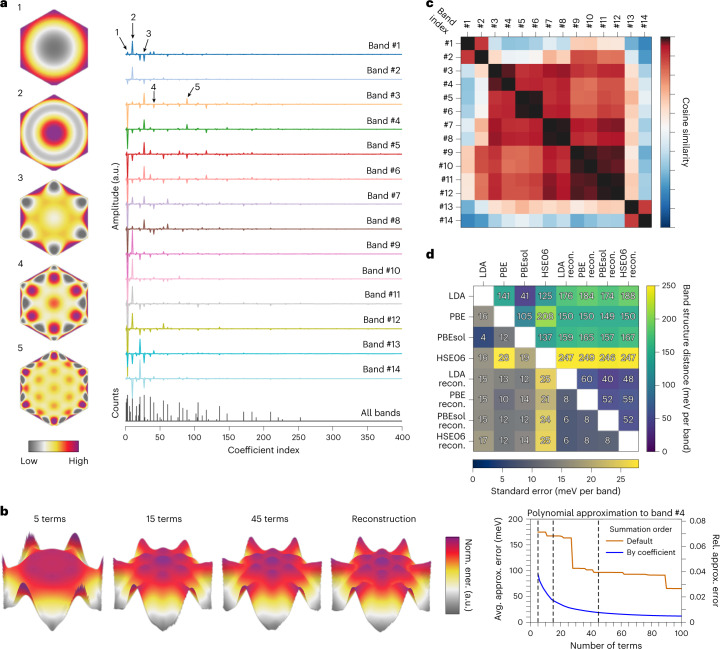
Digitization and comparison of WSe_2_ BSs. **a**, Decomposition of the 14 energy bands of WSe_2_ into hexagonal ZPs, with selected major terms displayed on the left. The zero spatial frequency term in the decomposition is subtracted for each band. The counts of large (>10^−2^ by absolute value) coefficients of all 14 bands are accumulated in the bottom row of the decomposition to illustrate their distribution; these decrease towards higher-order terms. **b**, Approximation of the shape (or dispersion) of the fourth energy band using 5, 15 and 45 hexagonal ZPs are compared with the reconstruction. The three approximated ones are indicated in the right figure by the vertical dashed lines intercepting the solid blue line. The errors in meV are calculated using equation (9) in Methods. **c**, Cosine similarity matrix for pairwise comparison of the reconstructed band dispersion in Fig. 2. The band indices follow those in Fig. 2d. **d**, Two-part similarity matrix showing BS distances (in the upper triangle) and their corresponding standard errors (in the lower triangle) between the computed and reconstructed BSs of WSe_2_. The abbreviation ‘LDA recon.’ denotes reconstruction with the LDA-level DFT BS as the initialization. Source data

The 14 reconstructed valence bands of WSe_2_ initialized by the local density approximation (LDA)-level DFT are shown in Fig. 2b–d and Supplementary videos. To globally compare the computed and reconstructed bands at a consistent resolution, we expand the BS in orthonormal polynomial bases^33^, which are global shape descriptors and unbiased by the underlying electronic detail. The geometric featurization of band dispersion allows multiscale sampling and comparison using coefficient (or feature) vectors^34^. We chose Zernike polynomials (ZPs) to decompose the 3D dispersion surfaces (Fig. 3 and Methods) because of their existing adaptations to various boundary conditions^35^.

In Fig. 3a,b, the band dispersions show generally decreasing dependence (seen from the magnitude of coefficients) on basis terms with increasing complexities (Fig. 3a), and the majority of dispersion is encoded into a subset of the terms (Fig. 3b). This observation implies that moderate smoothing may be applied to remove high-frequency features to improve the reconstruction in the case of limited-quality data (acquired without sufficient accumulation time), which is often unavoidable when materials exhibit vacuum degradation, or during experimental parameter tuning. The example in Fig. 3b and additional numerical evidence in Supplementary Fig. 14 illustrate the approximation capability of the hexagonal ZPs. These coefficients act as geometric fingerprints of the energy band dispersion, enabling the use of similarity or distance metrics (Methods) for their comparison^34^. In Fig. 3c, the positive cosine similarity confirms the strong shape (or dispersion) resemblance of the seven pairs of spin-split energy bands in the reconstructed BS of WSe_2_, and the low negative values, such as those for bands 1–2 and 13–14, reflect the opposite directions of their respective dispersion (Fig. 2d). These observations are consistent with the outcome obtained from DFT calculations (Supplementary Fig. 13).

### Computational metrics and performance

To quantify the computational advantages of the machine-learning-based reconstruction approach, we examine the outcome from diverse perspectives related to consistency, accuracy and cost. To assess the consistency of reconstruction in its entirety, we introduce a BS distance metric (Methods), invariant to the global energy shift frequently used to adjust the energy zero, to quantify the differences in band dispersion and the relative spacing between bands, which are the two major sources of variation between theories and experiments. The distance is calculated using the geometric fingerprints to bypass interpolation errors while reconciling the coordinate spacing difference between reconstructed and theoretical BSs, essential for differentiating BS data from heterogeneous sources in materials science databases^36,37^. The results in Fig. 3d refer to the valence BS of WSe_2_ discussed in this work, with the distances (Methods) and their spread (that is, standard errors) displayed in the upper and lower triangles, respectively. A high degree of consistency exists among the reconstructions (pairwise distance no larger than 60 ± 8 meV per band), regardless of the level of DFT calculation used for initialization, indicating the robustness of the probabilistic reconstruction algorithm, whereas the distances between the DFT calculations are much larger, both in energy shifts and their spread. As shown in Fig. 3d and Supplementary Fig. 5, the learning algorithm can effectively reduce the epistemic uncertainty^38^ between theories to obtain a consistent reconstruction.

**Fig. 4:**
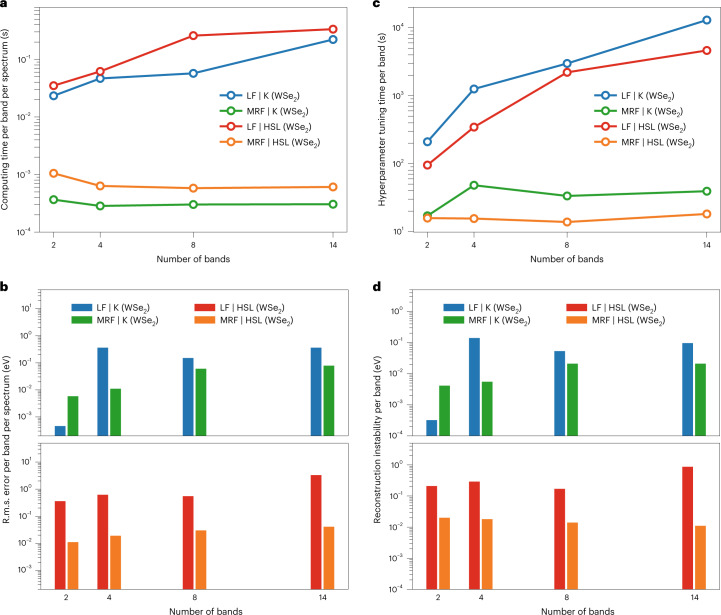
Performance evaluation on benchmarks. Visual summary of the benchmarking outcomes for BS reconstruction using normalized metrics that are able to compare across datasets. **a**,**b**, Computing time (**a**) and same-resolution r.m.s. error (reconstruction error) (**b**), both normalized to the per-band, per-spectrum level^40^. **c**,**d**, Hyperparameter tuning time (**c**) and reconstruction instability (s.d. of the residuals) (**d**), normalized to the per-band level. The methods used in reconstruction include pointwise line fitting (LF) and the MRF approach presented in this work, and the synthetic data are around the K-point and along the high-symmetry line (HSL) of the WSe_2_ BS. The benchmarks were run with synthetic datasets terminated at fixed energy ranges that contain the specified number of bands (2, 4, 8 and 14, the maximum band index in the dataset) shown in **a**–**d**. Source data

To demonstrate the computational advantage of the MRF reconstruction over traditional line-fitting methods, we benchmarked the outcome over selected regions in synthetic photoemission data. The regions are chosen based on their importance, and we limit the size to have a manageable computing time (about an hour on our computing cluster, at maximum, for a single run), determined by the slower method, and to allow for hyperparameter tuning, which requires tens of runs. The line-fitting approach uses the Levenberg–Marquardt least-squares optimization^39^ with bound constraints for multicomponent photoemission spectra composed of a series of lineshape functions. We used the benchmark established in ref. ^40^ for pointwise line fitting, employing high-performance computing and two synthetic datasets with known ground-truth dispersion, representing the local and global settings of the BS reconstruction problem (Supplementary Section 2.5). The synthetic data were based on a BS at the LDA-DFT level around the K-point and along the high-symmetry line of the Brillouin zone. To limit the hardware requirements, we used only distributed multicore-CPU computing for performance benchmarking. The estimated computing times are normalized to the per-band per-spectrum level^40^. The accuracy of the reconstruction is calculated using the same-resolution root-mean-squared (r.m.s.) error, and the (in)stability is quantified by the standard deviation (s.d.) of the residuals, which measures surface roughness^41^. The benchmarking results are compiled in Fig. 4 and Supplementary Table 2. They show that, compared with pointwise line fitting, the MRF reconstruction offers a considerable reduction in both normalized computing time and hyperparameter tuning time, while achieving consistently higher accuracy and stability in all but the two-band case. The combination of accuracy and stability in MRF reconstruction is due to the connectivity built into the prior, whereas in the pointwise fitting approach, information is not explicitly shared among neighbors. Because the number of bands reflects the complexity of the multicomponent spectra, near-constant normalized computing time and hyperparameter tuning time (Fig. 4a,b) in the MRF reconstruction, regardless of the number of bands (or spectral components), allow us to scale up the computation to datasets comprising 10^4^ to 10^5^ or more spectra. The substantial gain in computational efficiency is a result of the inherent divide-and-conquer strategy in our BS reconstruction problem formulation and the associated distributed optimization method in the algorithm design. Comparatively, the distributed pointwise fitting exhibits a quasi-linear computational scaling with respect to the number of bands. When hyperparameter tuning is taken into account, in practice it is only feasible for fitting small datasets with up to 10^3^ multicomponent spectra^40^.

### Extended use cases and applications

**Fig. 5:**
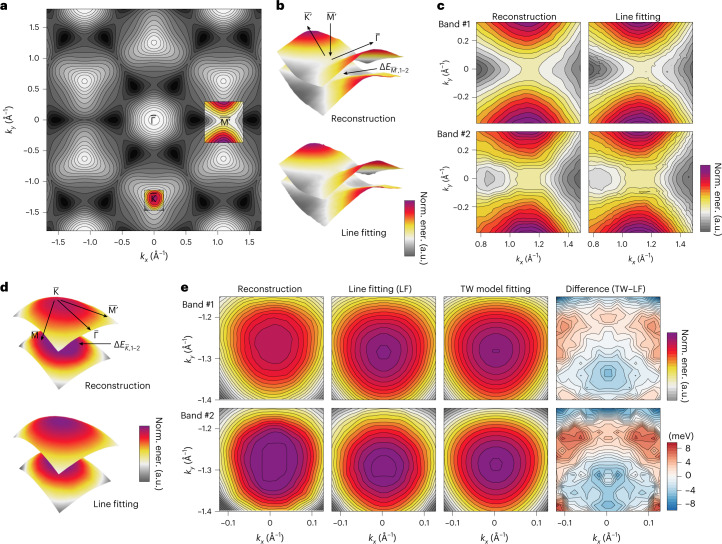
Local BS parameters of WSe_2_. **a**, The first valence band of 2*H*-WSe_2_, with constant-energy contours, from LDA-DFT calculation. The patches overlaid in color around high-symmetry points ${\overline{{{{\rm{K}}}}}}$ and ${\overline{{{{\rm{M}}}}}}\,^{{\prime} }$ are from reconstruction (with LDA-DFT as the initialization). **b**,**c**, Patch around the ${\overline{{{{\rm{M}}}}}}\,^{{\prime} }$-point, a saddle point in the dispersion surface, visualized in 3D (**b**) and 2D (**c**). The energy gap at ${\overline{{{{\rm{M}}}}}}\,^{{\prime} }$ due to SOC results in the energy difference ${{\Delta }}{E}_{{\overline{{{{\rm{M}}}}}}\,^{{\prime} },\,1-2}$. **d**,**e**, Patch around the ${\overline{{{{\rm{K}}}}}}$-point, the energy maximum of the valence band, visualized in 3D (**d**) and 2D (**e**). The SOC results in the energy gap ${{\Delta }}{E}_{\overline{{{{\rm{K}}}}},\,1-2}$. The outcome of fitting to a trigonal warping (TW) model around ${\overline{{{{\rm{K}}}}}}$ from a **k**⋅**p** theory model^28^ is shown in **e**. Source data

The band dispersions recovered from photoemission data are often examined locally near dispersion extrema. We show in Fig. 5 that, besides providing the global structural information, the reconstruction improves the robustness of traditional pointwise lineshape fitting in extended regions of the momentum space, when used as an initial guess, because BS calculations may exhibit appreciable momentum-dependent deviations from experimental data that prevent them from being a sufficiently good starting point. Pointwise fitting in turn acts as the refinement of local details not explicitly included in the probabilistic reconstruction model, which prioritizes efficiency. This sequential approach recovers large regions in the Brillouin zone at high energy resolution, without laborious hand-tuning of the fitting parameters per photoemission spectrum. Adopting this approach to WSe_2_, we first recovered a compendium of local BS parameters (Supplementary Table 4). The trigonal warping parameters of the first two valence bands around the ${\overline{{{{\rm{K}}}}}}$-point are 5.8 eV Å^3^ and 3.9 eV Å^3^, respectively, confirming the magnitude difference between these spin-split bands predicted by theory^28^. The warping signature extends further to high-energy bands. Dispersion fitting around the saddle point ${\overline{{{{\rm{M}}}}}}^{{\prime} }$ (and ${\overline{{{{\rm{M}}}}}}$) of the BS reveals that the gap opened by the spin–orbit interaction extends beyond it anisotropically on the dispersion surfaces, with the minimum gap at 338 meV, markedly larger than in the DFT results, which predict degeneracy^28^. We expect this observation to contribute to the spin-dependent optical absorption due to the association of the saddle point, in energy dispersion, with a van Hove singularity^28,42^.

**Fig. 6:**
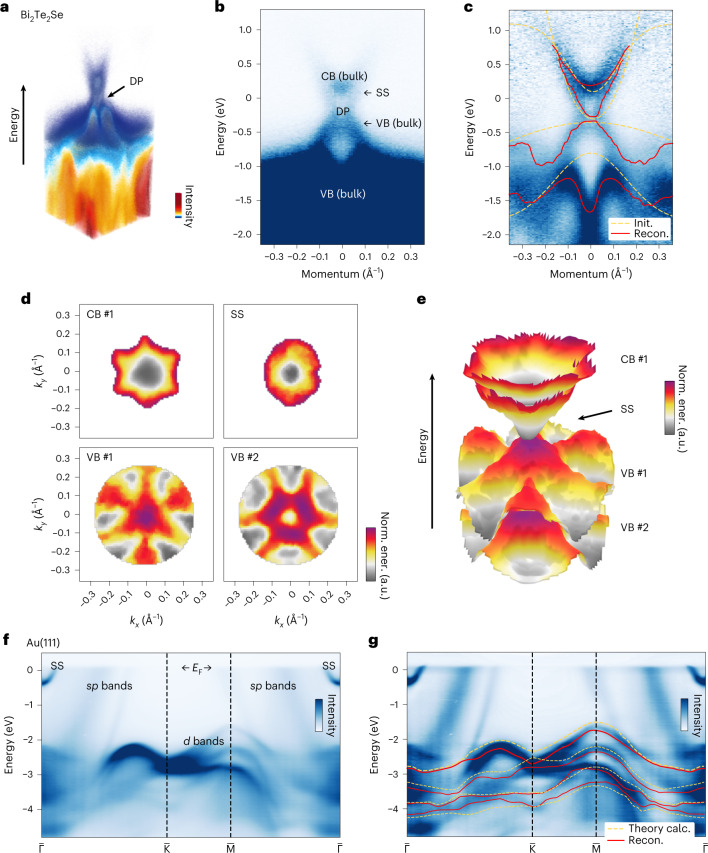
Band reconstruction for Bi_2_Te_2_Se and Au(111). **a**, 3D view of the photoemission band-mapping data of the topological insulator Bi_2_Te_2_Se around the Dirac point (DP). **b**, The energy bands near the DP are labeled in a 2D data slice through the DP. CB, conduction band; VB, valence band; SS, surface state. **c**, The outcome of reconstruction (after smoothing) is superimposed on the pre-processed data. **d**,**e**, Momentum-resolved reconstruction, shown in 2D (**d**) and 3D (**e**), where the color map represents the normalized energy values within each band. **f**, Experimental photoemission data for Au(111), shown with orbital character labels (*s*, *p*, *d*) of the energy bands, and the Fermi energy *E*_F_. **g**, Reconstruction of some of the *d* bands of Au(111), along with the theoretical calculations used for initialization. Source data

In addition to WSe_2_, we performed BS reconstruction on two other photoemission datasets for other classes of material. The first dataset is from bismuth tellurium selenide (Bi_2_Te_2_Se), a topological insulator, measured using the same laboratory photoemission set-up (Fig. 6a–e) as for the WSe_2_ dataset. Although we used only simple numerical functions (Gaussian and paraboloid) to initialize the MRF reconstruction, the outcome demonstrates correct discrete momentum–space symmetry and details of energy dispersion down to the concave-shaped hexagonal warping in the band energy contours around the Dirac point^43^. Four energy bands, including the two low-energy valence bands, a surface-state energy band, and a partially occupied conduction band, were recovered using our approach for Bi_2_Te_2_Se. The second is the bulk gold (Au) photoemission dataset measured at a synchrotron X-ray source (Fig. 6f,g). We used DFT calculations as the initialization to reconstruct four of the bulk energy bands, which are usually very challenging to extract by hand-tracing or parametric function-fitting, due in part to blurring (*k*_*z*_ dispersion) from the 3D characteristics of the electrons in the metallic bulk. Further discussions on these two materials and their band reconstructions are provided in Supplementary Section 3.

## Discussion

The reconstruction approach described here provides a quantitative connection between empirical band dispersion (${E}_{b}^{{{{\rm{emp}}}}}$) obtained from photoemission band mapping and the theoretical counterparts (${E}_{b}^{{{{\rm{theory}}}}}$) through various orders of momentum-dependent ‘perturbations’ (${{\Delta }}{E}_{b}^{(n)}$). The connection may be expressed as

3 $$\begin{array}{lll}{E}_{b}^{{{{\rm{emp}}}}}({{{\bf{k}}}},\,{{\varSigma }})&\approx &{E}_{b}^{{{{\rm{theory}}}}}({{{\bf{k}}}},\,{{\varSigma }})+{{\Delta }}{E}_{b}^{(0)}+{{\Delta }}{E}_{b}^{(1)}({{{\bf{k}}}},\,{{\varSigma }})+{{\Delta }}{E}_{b}^{(2)}({{{\bf{k}}}},\,{{\varSigma }})+...\\ &=&{E}_{b}^{{{{\rm{theory}}}}}({{{\bf{k}}}},\,{{\varSigma }})+\mathop{\sum}\limits_{n}{{\Delta }}{E}_{b}^{(n)}({{{\bf{k}}}},\,{{\varSigma }})\\ &=&{E}_{b}^{{{{\rm{theory}}}}}({{{\bf{k}}}},\,{{\varSigma }})+{{\Delta }}{E}_{b}({{{\bf{k}}}},{{\varSigma }}).\end{array}$$

In equation (3), *b* is the band index, *Σ* represents electron self-energy, the zeroth-order term (${{\Delta }}{E}_{b}^{(0)}$) means a rigid shift, and higher-order terms have increasing momentum-dependent nonlinearities. Our results here demonstrate that this formulation leads to practical band reconstruction, which recovers the accumulated perturbations (Δ*E*_*b*_) in equation (3) for every experimentally resolvable energy band. The outcome with current reconstruction accuracy and stability should assist interpretation of deep-lying bands, parametrizing multiband Hamiltonian models^44^. The data size reduction by over 5,000 times from 3D band-mapping data to geometric features vectors (Methods) facilitates database integration^37,45^.

Apart from the benefits, we want to outline three limitations of our reconstruction approach. First, the reconstruction approach does not work ab initio and requires knowing the number of energy bands, *N*_*b*_, as implicated by equation (3) for an indexed band (*b* = 1, 2, ..., *N*_*b*_). Although in simple datasets with up to several bands, *N*_*b*_ can be estimated using prior knowledge of the material or from visual inspection, correctly estimating *N*_*b*_ in complex datasets still requires calculated BSs. Second, when the electron self-energy modulation is substantial, separating the so-called bare-band dispersion (that is, single-particle dispersion) from the quasiparticle dispersion is needed to understand the material physics^46^. This requires re-evaluating the BS reconstruction concept and considering the full spectral function (Supplementary Section 1.1) explicitly to account for non-standard lineshapes. Nevertheless, the outcome of our current approach may act as a trial solution for disentangling the bare-band dispersion relation from the electron self-energy^46^. Because the local connectedness assumption in equation (2) remains largely valid, our reconstruction may still recover the quasiparticle dispersion. We demonstrate this in Supplementary Fig. 10 using simulated photoemission data with a kink anomaly, a strong modification of dispersion from electron self-energy^5,6^. Third, an appropriate initialization may be expensive or impossible to obtain, either due to the computational cost, if higher-level theories (such as DFT with hybrid functionals and GW) are required, or due to the complexity of the materials system, including undetermined microscopic interactions, sample defects or structural disorder, creating strong intensity blurring from *k*_*z*_ dispersion and so on. These scenarios will remain challenging for band reconstruction.

Besides our demonstrations, we anticipate additional use cases. These include (1) online monitoring^47^ of band-mapping experiments in the study of materials’ phase transitions^48^ or functioning devices^49^, where changes in atomic structure or carrier mobility are often accompanied by detectable changes in the electronic structure (including band dispersion), resulting in *I*(**k**, *E*, *t*) with time (*t*) dependence in addition to momentum (**k**) and energy. There is also (2) spatial mapping of BS variations for electronic devices via scanning photoemission measurements^50,51^, resulting in *I*(**k**, *E*, **x**) with spatial (**x**) dependence. In cases (1) and (2), a fast reconstruction and evaluation framework may be used in a feedback loop to steer or optimize experimental conditions. The next use case is (3) implementation of the reconstruction across various materials and to band-mapping data^7^ conditioned on external parameters, including temperature, photon energy, dynamical time delay, and spin as resolved quantities, which will generate comprehensive knowledge about the (non)equilibrium electronic structure of materials to benchmark theories. Moreover, the reconstruction method is (4) transferable to extracting the band dispersion of other quasiparticles (phonons^52^, polaritons^53^ and so on^54^) in periodic systems, given the availability of corresponding multidimensional datasets. Finally, (5) the analogy between band mapping and spatially resolved spectral imaging, which produces location-dependent spectra, or *I*(*x*, *y*, *E*) suggests that the reconstruction algorithm may find use in teasing out the spatial (*x*, *y*) variation of the spectral shifts, complementary to the outcome of clustering algorithms^55^.

The increasing amount of publicly accessible and reusable datasets from materials-science communities^45^ motivates future extensions to the model with other types of informative prior that account for the full complexity of the physical signal while maintaining computational efficiency. Overall, the multidisciplinary methodology provides an example of building next-generation high-throughput materials-characterization toolkits combining learning algorithms with physical knowledge^56^ to arrive at a comprehensive understanding of materials properties that has been unattainable so far.

## Methods

### Band-mapping measurements of WSe_2_

Multidimensional PES experiments were conducted with a laser-driven, high-harmonic-generation-based XUV light source^9^ operating at 21.7 eV and 500 kHz and a METIS 1000 (SPECS) momentum microscope featuring a delay-line detector coupled to a time-of-flight drift tube^8,57^. The experiment captures photoelectrons directly in their 3D coordinates, (*k*_*x*_, *k*_*y*_, *E*)^7,8^. Single-crystal samples of WSe_2_ (>99.995% pure) were purchased from HQ Graphene and were used directly for measurements without further purification. Before measurements, the WSe_2_ samples were attached to the Cu substrate with conductive epoxy resin (EPO-TEK H20E). The samples were cleaved by cleaving pins attached to the sample surface upon transfer into the measurement chamber, which operated at an ambient pressure of 10^−11^ mbar during photoemission experiments. No effect of surface termination was observed in the measured WSe_2_ photoemission spectra, similar to previous experimental observations^11,26^. For the valence-band-mapping experiments, the energy focal plane of the photoelectrons within the time-of-flight drift tube was set close to the top valence band. Although effects of sample degradation have been reported^27^ during the course of long-duration angular scanning in ARPES measurements, with our high-repetition-rate photon source^9^ and the fast electronics of the momentum microscope, band mapping of WSe_2_ achieves a sufficient signal-to-noise ratio for valence-band reconstruction within only tens of minutes of data acquisition, without the need for angular scanning and subsequent reconstruction from momentum–space slices.

### Data processing and reconstruction

The raw data, in the form of single-electron events recorded by the delay-line detector, were pre-processed using home-developed software packages^58^. The events were first binned to the (*k*_*x*_, *k*_*y*_, *E*) grid with dimensions of 256 × 256 × 470 to cover the full valence-band range in WSe_2_ within the projected Brillouin zone (PBZ), which amounts to a pixel size of ~0.015 Å^−1^ along the momentum axes and ~18 meV along the energy axis. The bin sizes are within the limits of the momentum resolution (<0.01 Å^−1^) and energy resolution (<15 meV) of the photoelectron spectrometer^59^.

Data binning was carried out in conjunction with the necessary lens distortion correction^60^ and calibrations, as described in ref. ^58^. The outcome provided a sufficient level of granularity in momentum space to resolve the fine features in band dispersion while achieving higher signal-to-noise ratio than when using single-event data directly. Afterwards, we applied intensity symmetrization to the data along the six-fold rotation symmetry and mirror symmetry axes^11^ of the photoemission intensity pattern in (*k*_*x*_, *k*_*y*_) coordinates, followed by contrast enhancement using the multidimensional extension of the contrast limited adaptive histogram equalization (MCLAHE) algorithm, where the intensities in the image are transformed by a look-up table built from the normalized cumulative distribution function of local image patches^29^. Finally, we applied Gaussian smoothing to the data along the *k*_*x*_, *k*_*y*_ and *E* axes with s.d. of 0.8, 0.8 and 1 pixels (or ~0.012 Å^−1^, 0.012 Å^−1^, and 18 meV), respectively.

After data pre-processing, we sequentially reconstructed every energy band of WSe_2_ from the photoemission data using the MAP approach described in the main text. The reconstruction requires tuning of three hyperparameters: (1) momentum scaling and (2) the rigid energy shift to coarse-align the computed energy band, for example, from DFT, to the photoemission data, and (3) the width of the NN Gaussian prior (*η* in equation (2)). Hyperparameter tuning is also carried out individually for each band to adapt to a specific environment. An example of hyperparameter tuning is given in Supplementary Fig. 4. The MAP reconstruction method involves optimization of the band-energy random variables, ${\{{\tilde{E}}_{i,\,j}\}}$, to maximize the posterior probability, ${p}={p}(\{{\tilde{E}}_{i,\,j}\})$, or to minimize the negative log-probability loss function, ${{{\mathcal{L}}}}:= {-\log p}$, obtained from equation (2) as is used in our actual implementation:

4 $${{{\mathcal{L}}}}(\{{\tilde{E}}_{i,\,j}\})={-\mathop{\sum}\limits_{i,\,j}\log I({k}_{x,\,i},\,{k}_{y,\,j},\,{\tilde{E}}_{i,\,j})+\mathop{\sum}\limits_{(i,\,j),\,(l,\,m)| {{{\rm{NN}}}}}\frac{{({\tilde{E}}_{i,\,j}-{\tilde{E}}_{l,\,m})}^{2}}{2{\eta }^{2}}+{{{\rm{const.}}}}}$$

We implemented the optimization using a parallelized version of the iterated conditional mode^61^ method in TensorFlow^62^ to run on multicore computing clusters and GPUs. The parallelization involves a checkerboard coloring scheme (or coding method) of the graph nodes^63^ and subsequent hierarchical grouping of colored nodes, which allows alternating updates on different subgraphs (that is, subsets of the nodes) of the MRF during optimization. Typically, the optimization process in the reconstruction of one band converges within and therefore is terminated after 100 epochs, which takes ~7 s on a single NVIDIA GTX980 GPU for the above-mentioned data size. Details on the parallelized implementation are provided in Supplementary Section 1. In addition, because symmetry information is not explicitly included in the MRF model, the reconstructed bands generally require further symmetrization, such as refinement or post-processing, to be ready for database integration.

We have described our approach of using BS calculations to initialize the MAP optimization as a warm start. The term ‘warm start’ in the context of numerical optimization generally refers to the initialization of an optimization using the outcome of an associated but more solvable problem (for example, a surrogate model) obtained beforehand that yields an approximate answer, instead of starting from scratch (cold start). Warm-starting an optimization improves the effective use of prior knowledge and its convergence rate^39^. In the current context, we regard the BS reconstruction from photoemission band-mapping data as the optimization problem to warm start, and the outcome from an electronic-structure calculation can produce a sufficiently good approximate to the solution of the optimization problem. For WSe_2_, straightforward DFT calculations with semi-local approximation (which in itself involves explicit optimizations such as geometric optimization of the crystal structures) are sufficient, but our approach is not limited to DFT. Therefore, the use of ‘warm start’ in our application is conceptually well-aligned with the origin of the term.

To validate the MAP reconstruction algorithm in a variety of scenarios, we used synthetic photoemission data where the nominal ground-truth BSs are available. The BSs are constructed using analytic functions, model Hamiltonians or DFT calculations. The initializations are generated by tuning the numerical parameters used to generate the ground-truth BSs. The procedures and results are presented in Supplementary Section 2. In simple cases, such as single or well-isolated bands, the reconstruction yields a close solution to the ground truth, even with a flat band initialization. In the more general multiband scenario with congested bands and band crossings (or anti-crossings), an approximate dispersion (or shape) of the band and the crossing information is required in the initialization (warm start) to converge to a realistic solution. We further tested the robustness of the initializations by (1) scaling the energies of the ground truth and (2) using DFT calculations with different exchange-correlation (XC) functionals, to capture sufficient variability of available BS calculations in the real world. We quantify the variations in the initializations and the performance of the reconstruction using the average error (equation (9) or Fig. 4b), calculated with respect to the ground truth. Among the different numerical experiments, we find that the optimization converges consistently to a set of bands that better match the experimental data than the initialization. This is manifested in the fact that the average errors of the initializations are reduced to a similar level in the corresponding reconstruction outcomes, a trend seen over all bands, regardless of their dispersion. In the synthetic data with an energy spacing of ~18 meV, the average error in the reconstruction is on the order of 40–50 meV for each band, which amounts to an average inaccuracy of <3 bins along the energy dimension at a momentum location. The inaccuracy is, however, dependent on the bin sizes used in pre-processing and the fundamental resolution in the experiment. We have made the code for the MAP reconstruction algorithm and the synthetic data generation publicly accessible from the online repository Fuller^64^ for broader applications.

### Visualization strategies

Band-mapping and BS data contain unique multidimensional data structures in materials science that are often presented with specific visualizations motivated by the underlying solid-state physics and symmetry properties. In this Article we select a fixed set of 2D and 3D visualization techniques to illustrate their links and allow comparison with other photoemission studies of the same materials. Typically, ARPES data^6^ of the form *I*(*E*, *k*) are sampled and visualized along a particular path (the k-path^65^) in momentum space^26,27^, where only specific high-symmetry positions are labeled with capital letters^3^. A canonical k-path exists for each space group symmetry setting^65^. Photoemission band mapping generates datasets with a dimensionality of three or higher, and often contains a lower symmetry (in intensity *I*) as a result of the photoemission matrix elements^20^ and the experimental conditions. These factors lead to more flexibility in data representation^58^ and motivate the use of alternate k-paths that capture the complexity of the photoemission spectra. In Fig. 1c–f for WSe_2_ and Fig. 6a–c for Bi_2_Te_2_Se, we combine 3D volumetric rendering and 2D k-path views to illustrate both the data symmetry and the intensity modulations present in the data.

To visualize the band dispersion surfaces, *E*_*b*_(*k*_*x*_, *k*_*y*_) (*b* = 1, 2, ...), we combine 3D stacked surfaces and 2D image sequences, as exemplified in Fig. 2b,d for WSe_2_ and Fig. 6d,e for Bi_2_Te_2_Se. This paired visualization approach balances the strengths and shortcomings of different viewpoints to achieve a comprehensive representation of the data type. The 3D stacked surface representation highlights the entirety and complexity of the data, but often contains occluded regions imperceptible from a fixed viewing direction. The 2D-image-sequence representation includes all energy dispersion information, yet loses the inter-relationship on the energy scale between energy bands, which matters in the event of (anti)crossings. In combining these two approaches, we typically choose the same color map and scale to maintain referenceability between the two representations. For each energy band, the full color scale is used to cover its energy range, becoming the normalized energy (norm. ener.) scale, which illustrates the local detail of the dispersion that otherwise may be hard to discern.

### BS calculations

Electronic BSs were calculated within (generalized) DFT using the LDA^66,67^, the generalized-gradient approximation (GGA-PBE)^68^ and GGA-PBEsol^69^), and the hybrid XC functional HSE06^70^, which incorporates a fraction of the exact exchange. All calculations were performed with the all-electron, full-potential numeric-atomic orbital code, FHI-aims^71^. They were conducted for the geometries obtained by fully relaxing the atomic structure with the respective XC functional to keep the electronic and atomic structures consistent. SOC was included in a perturbational fashion^72^. The momentum grid used for the calculation was equally sampled with a spacing of 0.012 Å^−1^ in both *k*_*x*_ and *k*_*y*_ directions, which covers the irreducible part of the first Brillouin zone at *k*_*z*_ = 0.35 Å^−1^, estimated using the inner potential of WSe_2_ from a previous measurement^11^. The calculated BS is symmetrized to fill the entire hexagonal Brillouin zone used to initialize the BS reconstruction and synthetic data generation. We note here that, for MAP reconstruction, the momentum grid size used in the theoretical calculations (such as DFT at various levels as used here) need not be identical to that of the data (or instrument resolution), and in such cases an appropriate upsampling (or downsampling) should be applied to the calculation to match the momentum resolution. Further details are presented in Supplementary Section 4.

### BS informatics

The shape feature-space representation of each electronic band is derived from the decomposition

5 $${E}_{b}({{{\bf{k}}}})=\mathop{\sum}\limits_{l}{a}_{l}{\phi }_{l}({{{\bf{k}}}})={{{\bf{a}}}}\cdot {{{\bf{\Phi }}}}.$$

Here, **k** = (*k*_*x*_, *k*_*y*_) represents the momentum coordinate, *E*_*b*_(**k**) is the single-band dispersion relation (for example, the dispersion surface in 3D), and *a*_*l*_ and *ϕ*_*l*_(**k**) are the coefficient and its associated basis term, respectively. The latter are grouped separately into the feature vector, **a** = (*a*_1_, *a*_2_, ...) and the basis vector, **Φ** = (*ϕ*_1_, *ϕ*_2_, ...). The orthonormality of the basis is guaranteed within the PBZ of the material:

6 $${\int}_{{{{\bf{k}}}}\in {{{\varOmega }}}_{{{{\rm{PBZ}}}}}}{\phi }_{m}({{{\bf{k}}}}){\phi }_{n}({{{\bf{k}}}}){\rm{d}}{{{\bf{k}}}}={\delta }_{mn}.$$

For the hexagonal PBZ of WSe_2_, the basis terms are hexagonal ZPs constructed using a linear combination of the circular ZPs via Gram–Schmidt orthonormalization within a regular (that is, equilateral and equiangular) hexagon^35^. A similar method can be used to generate the ZP-derived orthonormal basis adapted to other boundary conditions^35^. The representation in feature space^34^ provides a way to quantify the difference (or distance) *d* between energy bands or BSs at different resolutions or scales, without additional interpolation. To quantify the shape similarity between energy bands *E*_*b*_ and ${E}_{{b}^{{\prime} }}$, we calculate the cosine similarity using the feature vectors

7 $${d}_{\cos }({E}_{b},{E}_{{b}^{{\prime} }})=\frac{{{{\bf{a}}}}\cdot {{{{\bf{a}}}}}^{{\prime} }}{| {{{\bf{a}}}}| \cdot | {{{{\bf{a}}}}}^{{\prime} }| },$$

where the cosine similarity is bounded within [−1, 1], with a value of 0 describing orthogonality of the feature vectors and a value of 1 and −1 describing parallel and anti-parallel relations between them, respectively, both indicating high similarity. The use of cosine similarity in feature space allows comparison of dispersion while being unaffected by their magnitudes. In comparing the dispersion between single energy bands using equation (7), the first term in the polynomial expansion, or the hexagonal equivalent of the Zernike piston^73^, is discarded as it only represents a constant energy offset (with zero spatial frequency) instead of dispersion, which is characterized by a combination of finite and nonzero spatial frequencies.

The electronic BS is a collection of energy bands ${E}_{B}=\{{E}_{{b}_{i}}\}$ (*i* = 1, 2, ...). To quantify the distance between two BSs, ${E}_{{B}_{1}}=\{{E}_{{b}_{1,\,i}}\}$ and ${E}_{{B}_{2}}=\{{E}_{{b}_{2,\,i}}\}$, containing the same number of energy bands while ignoring their global energy difference, we first subtract the energy grand mean (that is, the mean of the energy means of all bands within the region of the BS for comparison). We then compute the Euclidean distance, or the *ℓ*^2^-norm, for the *i*th pair of bands, *d*_*b*, *i*_:

8 $${d}_{b,\,i}({E}_{{b}_{1,\,i}},{E}_{{b}_{2,\,i}})=\parallel {{\tilde{{{{\bf{a}}}}}}_{1,\,i}-{\tilde{{{{\bf{a}}}}}}_{2,\,i}\parallel }_{2}=\sqrt{\mathop{\sum}\limits_{l}{({\tilde{a}}_{1,\,il}-{\tilde{a}}_{2,\,il})}^{2}}.$$

Here, ${\tilde{{{{\bf{a}}}}}}$ denotes the feature vector after subtracting the energy grand mean, so that any global energy shift is removed. We define the BS distance as the average distance over all *N*_*b*_ pairs of bands, or ${d}_{B}({E}_{{B}_{1}},\,{E}_{{B}_{2}})$ = ${\mathop{\sum }\nolimits_{i}^{{N}_{b}}{d}_{b,\,i}({E}_{{b}_{1},\,i},{E}_{{b}_{2},\,i})/{N}_{b}}$. The values of ${d}_{B}({E}_{{B}_{1}},\,{E}_{{B}_{2}})$ are shown in the upper triangle of Fig. 3d and their corresponding standard errors (over the 14 valence bands of WSe_2_) in the lower triangle. The distance in equation (8) is independent of basis and allows energy bands calculated on different resolutions or from different materials with the same symmetry (for example, differing only by Brillouin zone size) to be compared.

We use same-resolution error metrics to evaluate the approximation quality of the expansion basis and to quantify the reconstruction outcome with a known ground-truth BS. Specifically, we define the average approximation error (with energy unit), *η*_avg_, for each energy band using the energy difference at every momentum location:

9 $${\eta }_{{{{\rm{avg}}}}}({E}_{{{{\rm{approx}}}}},{E}_{{{{\rm{recon}}}}})=\sqrt{\frac{1}{{N}_{k}}\mathop{\sum}\limits_{{{{\bf{k}}}}\in {{{\varOmega }}}_{{{{\rm{PBZ}}}}}}{({E}_{{{{\rm{approx}}}},\,{{{\bf{k}}}}}-{E}_{{{{\rm{recon}}}},\,{{{\bf{k}}}}})}^{2}},$$

where *N*_*k*_ is the number of momentum grid points and the summation runs over the PBZ. In addition, we construct the relative approximation error, *η*_rel_, following the definition of the normwise error^74^ in matrix computation:

10 $${\eta }_{{{{\rm{rel}}}}}({E}_{{{{\rm{approx}}}}},\,{E}_{{{{\rm{recon}}}}})=\frac{{\left\Vert {E}_{{{{\rm{approx}}}}}-{E}_{{{{\rm{recon}}}}}\right\Vert }_{2}}{{\left\Vert {E}_{{{{\rm{recon}}}}}\right\Vert }_{2}}.$$

Equations (9) and (10) are used to compute the curves in Fig. 3b as a function of the number of basis terms included in the approximation. The relevant code for the representation using hexagonal ZPs and the computation of the metrics is also accessible in the public repository Fuller^64^.

### Data reduction

The raw data and intermediate results are stored in the HDF5 format^58^. The file sizes quoted here for reference are calculated from storage as double-precision floats or integers (for indices). The photoemission band-mapping data of WSe_2_ (256 × 256 × 470 bins) have a size of ~235 MB (240,646 kB) after binning from single-event data (7.8 GB or 8,176,788 kB). The reconstructed valence bands at the same resolution occupy ~3 MB (3,352 kB) in storage, and the size further decreases to 46 kB when we store the shape feature vector associated with each band. If only the top-100 coefficients (ranked by the absolute values of their amplitudes) and their indices in the feature vectors are stored, the data amounts to 24 kB. For the case of WSe_2_, the top-100 coefficients can approximate the band dispersion with a relative error (equation (10)) of <0.8% for every energy band, as shown in Supplementary Fig. 14.

### Supplementary information

Supplementary Information Supplementary Figs. 1–14, Tables 1–4, derivations of formulae, extended numerical validations and discussion.

Supplementary Video 1 Left side shows the position of the cut viewed from the first projected Brillouin zone of WSe_2_. Right side shows the corresponding 2D cut in (*k*_*y*_, *E*) coordinates from volumetric band mapping data, overlaid with the DFT calculation performed at the LDA level (LDA-DFT), used to initialize the reconstruction, and the resulting 14 reconstructed valence bands.

Supplementary Video 2 Left side shows the position of the cut viewed from the first projected Brillouin zone of WSe_2_. Right side shows the corresponding 2D cut in (*k*_*x*_, *E*) coordinates from volumetric band mapping data, overlaid with the DFT calculation performed at the LDA level (LDA-DFT), used to initialize the reconstruction, and the resulting 14 reconstructed valence bands.

Supplementary Video 3 The video explores the reconstructed valence bands from photoemission band mapping data on WSe_2_ using LDA-level DFT calculation as the initialization. It illustrates the generation of an exploded view of the bands from the original reconstruction, the bands viewed collectively from different angles and the individual view of each band.

### Source data

Source Data Fig. 1 Numerical data contained in Fig. 1c–f.

Source Data Fig. 2 Numerical data contained in Fig. 2c,d.

Source Data Fig. 3 Numerical data contained in Fig. 3.

Source Data Fig. 4 Numerical data contained in Fig. 4.

Source Data Fig. 5 Numerical data contained in Fig. 5c,e.

Source Data Fig. 6 Numerical data contained in Fig. 6b–d,f,g.

## Data Availability

The electronic-structure calculations for WSe_2_ are available from the NOMAD repository (10.17172/NOMAD/2020.03.28-1)^75^. The raw and processed photoemission datasets used in this work for WSe_2_ (10.5281/zenodo.7314278)^76^, Bi_2_Te_2_Se (10.5281/zenodo.7317667)^77^ and Au(111) (10.5281/zenodo.7305241 including DFT calculation)^78^ are available on Zenodo. Source data are provided with this paper.

## Code availability

The code developed for band structure reconstruction, including examples, is available on GitHub (https://github.com/mpes-kit/fuller)^79^.
