# Supplementary material for: A machine learning route between band mapping and band structure
Source: Nat Comput Sci. 2022 Dec 30;3(1):101–14. doi: 10.1038/s43588-022-00382-2 (PMC10766556; doi:10.1038/s43588-022-00382-2)
Supplement: Supplementary file 1 — Supplementary Figs. 1–14, Tables 1–4, derivations of formulae, extended numerical validations and discussion. [file 43588_2022_382_MOESM1_ESM.pdf]

nature computational science

Resource https://doi.org/10.1038/s43588-022-00382-2

# A machine learning route between band mapping and band structure

In the format provided by the authors and unedited

# Contents


**1 Band structure reconstruction** **2**

1.1 Physical foundations . . . 2

1.2 Markov random field modeling . . . 3

1.3 Optimization procedure . . . 7

1.4 Hyperparameter tuning . . . 11

1.5 Reconstructions using different theories as initializations . . . 16

**2 Generation of and validation on synthetic data** **16**

2.1 Generation of band structure data . . . 17

2.2 Initialization tuning . . . 17

2.3 Approximate generation of photoemission data . . . 19

2.4 Validation of the reconstruction algorithm . . . 20

2.5 Computational benchmarks . . . 26

**3 Reconstruction for other datasets** **30**

3.1 Near-gap electronic bands of a topological insulator ($Bi_2Te_2Se$) . . . 30

3.2 Bulk electronic bands of gold (Au) . . . 31

3.3 Reconstructing the kink anomaly . . . 32

**4 Band structure calculations** **34**

4.1 DFT calculations . . . 34

4.2 Brillouin zone tiling . . . 37

**5 Band structure informatics** **40**

5.1 Global structure descriptors . . . 40

5.2 Local structure descriptors . . . 41

# 1 Band structure reconstruction

## 1.1 Physical foundations

The three quantities of common interest for the interpretation of photoemission spectra are (1) the bare band energy, $\epsilon_{\mathbf{k}}$, (2) the complex-valued electron self-energy, $\Sigma(\mathbf{k}, E) = \mathrm{Re}\Sigma(\mathbf{k}, E) + i\mathrm{Im}\Sigma(\mathbf{k}, E)$, and (3) the transition matrix elements connecting the final $(f)$ and initial $(i)$ electronic states, $M_{f,i}(\mathbf{k}, E)$. An established interface between theory and experiment for quantitating and interpreting the photoemission signal is the formalism of an experimental observable: the single-particle spectral function [5, 80], $A(\mathbf{k}, E)$. For a single energy band of a many-body electronic system,

$$A(\mathbf{k}, E) = \frac{1}{\pi} \frac{\mathrm{Im}\Sigma(\mathbf{k}, E)}{[E - \epsilon_{\mathbf{k}} - \mathrm{Re}\Sigma(\mathbf{k}, E)]^2 + [\mathrm{Im}\Sigma(\mathbf{k}, E)]^2}. \tag{11}$$

Within this framework, the band loci of the photoemission (or quasiparticle) band structure (BS), $b(\mathbf{k}, E) = \epsilon_{\mathbf{k}} + \mathrm{Re}\Sigma(\mathbf{k}, E)$, correspond to the bare band dispersion modulated by the real part of the electron self-energy, and they occupy the local maxima of the spectral function evaluated at different momenta. However, in the photoemission process, the intensity counts registered by the detector are modulated by the transition matrix elements [20], the Fermi-Dirac occupation function, $f_{\mathrm{FD}}(E)$, and the resolution of the measuring instrument, $G(E, \sigma_E, \sigma_{\mathbf{k}})$, typically a multidimensional Gaussian function. This leads to the expression of the photoemission intensity, $I(\mathbf{k}, E)$, registered on an energy- and momentum-resolved detector,

$$I(\mathbf{k}, E) \propto |M_{f,i}(\mathbf{k}, E)|^2 f_{\mathrm{FD}}(E) A(\mathbf{k}, E) \otimes G(E, \sigma_E, \sigma_{\mathbf{k}}). \tag{12}$$

For a multiband electronic structure, band mapping measurements, in principle, have access to the spectral functions of at least all valence bands. The photoemission intensities are combined

in summation to form the multiband (MB) counterpart of the single-band formula.

$$I_{\mathrm{MB}}(\mathbf{k},E)=\sum_j I_j(\mathbf{k},E)\propto\sum_j |M_{f_j,i_j}(\mathbf{k},E)|^2 f_{\mathrm{FD}}(E)A_j(\mathbf{k},E)\otimes G(E,\sigma_E,\sigma_{\mathbf{k}}) \tag{13}$$

$$\sim\sum_j A_j(\mathbf{k},E)\otimes G(E,\sigma_E,\sigma_{\mathbf{k}}),\quad (\mathrm{when}|M_{f_j,i_j}(\mathbf{k},E)|\to 1, f_{\mathrm{FD}}(E)\to 1). \tag{14}$$

The condition $f_{\mathrm{FD}}(E)\to 1$ applies to valence bands, while $|M_{f_j,i_j}(\mathbf{k},E)|\to 1$ may be achieved through nonlinear intensity normalization or contrast enhancement in data processing. The expression of the multiband photoemission intensity in Eqs. (13)-(14) provides the physical foundation and inspiration for the approximate generation of band mapping data (see Supplementary Section 2) that we employ to validate the reconstruction algorithm introduced in this work.

## 1.2 Markov random field modeling

The Markov random field (MRF) model for the photoemission band structure in photoemission band mapping data can be constructed similarly for data in multiple dimensions. In traditional angle-resolved photoemission spectroscopy (ARPES), photoemission intensities are measured in the $(k,E)$ coordinates, the proximity of the momentum positions in the band structure can be modeled using an MRF composed of a 1D chain of random variables as shown in Supplementary Fig. 1a. Band mapping data in $(k_x,k_y,E)$ coordinates, as described in the main text, can be modelled using a 2D MRF. In addition, the algorithm can be extended to higher dimensions involving coordinates beyond energy and momenta. For example, time-resolved photoemission data recorded in $(k_x,k_y,E,t)$ coordinates can be modelled using a 3D MRF as shown in Supplementary Fig. 1c. In the following, we provide a brief introduction to the theory underlying MRF and provide a simplified derivation of the 2D MRF model introduced in the main text.

Deriving the MRF amounts to determining the joint distribution of the random variables associated with its graphical representation. In probabilistic graphical model theory [81], a

graph is constructed from the fundamental components called cliques. Each clique $C$ of a graph is a subset of nodes that shares an edge with another node in $C$, with the total number of nodes in $C$ defined as its size. The MRFs in Supplementary Fig. 1a-c that model the photoemission data are built out of cliques of sizes 1–2 shown in Supplementary Fig. 1d. Although larger cliques can be constructed similarly [81], their parent graphical models are described by more complex joint distributions with drastically higher computational costs in optimization, therefore are not used in our MRFs. Mathematically, each clique is represented by a so-called potential function, $\psi_C$, which is used to derive the joint distribution that characterizes the MRF. The potential function only depends on the node configuration in the cliques, $\mathbf{X}_C$, and satisfies $\psi_C(\mathbf{X}_C) \geqslant 0$. According to the Hammersley-Clifford theorem [81–83], the joint distribution of a vector of random variables, $\mathbf{X}$, can be written in the factorized form,

$$p(\mathbf{X}) = \frac{1}{Z} \prod_{C \in \mathcal{C}} \psi_C(\mathbf{X}_C). \tag{15}$$

Here, $\mathcal{C}$ is the set of all cliques in the graph, and the partition function $Z$ is a normalization constant given by

$$Z = \sum_{\mathbf{X}} \prod_{C \in \mathcal{C}} \psi_C(\mathbf{X}_C).$$

The graphical representation of the MRFs relevant to this work are rectangular grids shown in Supplementary Fig. 1. The respective potential functions of the size-1 and size-2 cliques are interpreted as the likelihood and prior of the probabilistic graphical model, respectively. To cast the band structure reconstruction problem into this framework, we assign the band energies as the random variables in the model, and the potential function of each node (size-1 clique) as the (preprocessed) photoemission intensity at the respective grid position. For simplicity and computational efficiency, this formulation doesn't explicitly account for the intensity modulations described in Eq. (12) and preprocessing steps are required to neutralize their effects. The continuity assumption (i.e. no sharp jump) of the band energies along momentum directions

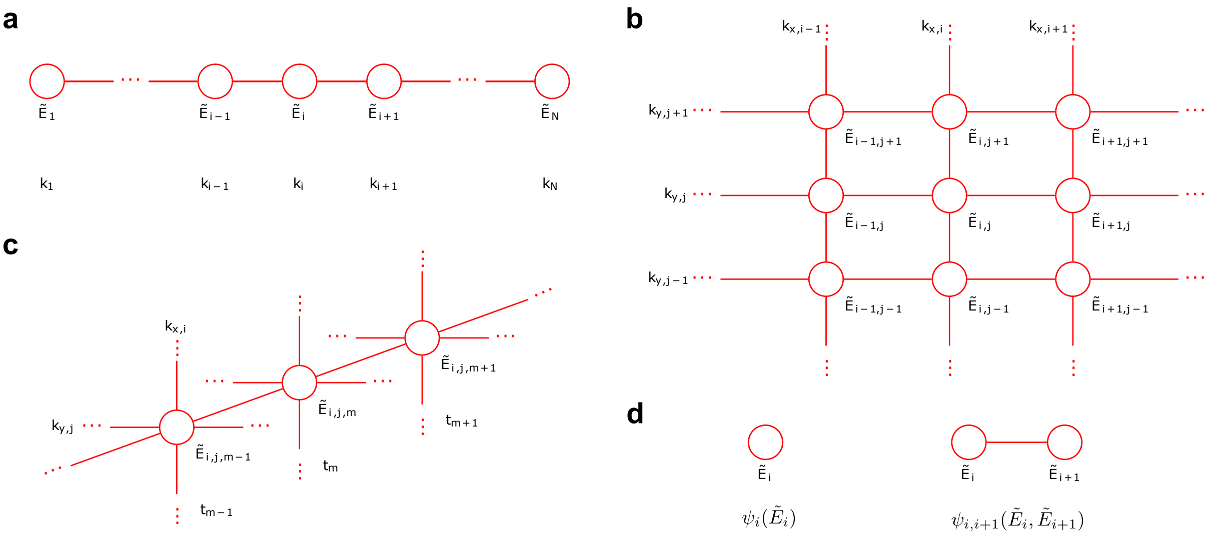


Supplementary Figure 1: **Examples of the MRF models for photoemission spectroscopy data**. **a**, 1D MRF model for data in $(k, E)$ coordinates, represented as a chain of random variables $\tilde{E}_i$. $N$ is the number of measured momentum values. **b**, 2D MRF model of photoemission data in $(k_x, k_y, E)$ coordinates as introduced and demonstrated for use in the main text, with the random variables $\tilde{E}_{i,j}$ connected on two dimensions $k_x$ and $k_y$. **c**, 3D MRF model for time- and momentum-resolved photoemission spectroscopy data in $(k_x, k_y, E, t)$ coordinates. The random variables $\tilde{E}_{i,j,m}$ are first connected in the graph to the neighboring momentum positions as in **b**, then subsequently along the neighboring time points. The time variable in **c** may also be replaced with other variables without changes in the structure of the graphical model. In **a**-**c**, the MRFs are constructed using components (cliques) with sizes 1 (left) and 2 (right) in **d**, with their respective potential functions written below the illustrations.

means that the potential function of size-2 cliques can be represented by a Gaussian on adjacent momentum grid positions. Intuitively, this means that the closer the two adjacent energies is, the more probable they are the actual band loci, and *vice versa*.

In the 1D case (see Supplementary Fig. 1a), the potential function of each node (containing one band energy random variable $\tilde{E}_i$) is given by

$$\psi_i(\tilde{E}_i) = \tilde{I}(k_i, \tilde{E}_i), \tag{16}$$

where $\tilde{I}$ is the photoemission intensity after preprocessing. The potential function of two connected nodes (describing the similarity between two neighboring band energy random variables)

is given by

$$\psi_{j,j+1}(\tilde{E}_j, \tilde{E}_{j+1}) = \exp\left[-\frac{(\tilde{E}_j - \tilde{E}_{j+1})^2}{2\eta^2}\right]. \tag{17}$$

Plugging Eqs. (16)-(17) into Eq. (15) yields

$$\begin{aligned} p(\tilde{E}_1, ..., \tilde{E}_N) =& \frac{1}{Z}\prod_{i=1}^{N}\psi_i(\tilde{E}_i)\cdot\prod_{j=1}^{N-1}\psi_{j,j+1}(\tilde{E}_j, \tilde{E}_{j+1}) \\ =& \frac{1}{Z}\prod_{i=1}^{N}\tilde{I}(k_i, \tilde{E}_i)\cdot\prod_{j=1}^{N-1}\exp\left[-\frac{\left(\tilde{E}_j - \tilde{E}_{j+1}\right)^2}{2\eta^2}\right] \end{aligned} \tag{18}$$

as the joint distribution of the 1D MRF, with $N$ being the total number of momentum grid points. Analogously, we can derive the joint distribution of the 2D MRF as given in the main text, and that for the 3D MRF in the $(k_x, k_y, E, t)$ coordinates is

$$p(\{\tilde{E}_{i,j,m}\}) = \frac{1}{Z}\prod_{i,j,m}\tilde{I}(k_{x,i}, k_{y,j}, t_m, \tilde{E}_{i,j,m})\cdot\prod_{(i,j,m),(l,o,q)|\mathrm{NN}}\exp\left[-\frac{(\tilde{E}_{i,j,m} - \tilde{E}_{l,o,q})^2}{2\eta^2}\right].$$

The MRF models in different dimensions discussed here follow the same Bayesian interpretation as the 2D MRF (Eq. (1) in the main text).

In practice, a 4D dataset of the kind, $I(E, k_x, k_y, k_z)$, and comparable spacing along the momentum dimensions ($\Delta k_x \sim \Delta k_y \sim \Delta k_z$) should be treated together to best use the connectivity encoded in the structured prior of the Markov random field model, which becomes a 3D grid of random variables that accounts for the connectedness along the momentum directions. When the fourth dimension (such as $k_z$) in the data is not sampled as densely as the other dimensions ($\Delta k_x \sim \Delta k_y \ll \Delta k_z$), which may be the case for synchrotron-based photoemission instruments (resulting in 3.5 D or quasi-4D datasets), the dataset can also be treated individually per scanned photon energy, since the local connectedness assumption along the third momentum direction is no longer retained.

## 1.3 Optimization procedure

Optimization of the MRF model is a local minima-finding process [81]. The following procedures are described using the 2D MRF in the main text as an example, but the approach can be extended to arbitrary dimensions. Due to the large number of random variables ($\sim 10^4$ for the 2D MRF in the main text) and their complex dependence structure in the MRF, we solved it numerically using iterated conditional mode (ICM) [61] procedure and implemented with efficient parallelization schemes, including the coding method and the hierarchical grouping of random variables. Next, we discuss the motivations and clarify the details of these three aspects. We provide the associated pseudocode in Algorithm 1.

**1. Iterated conditional mode**: Originally developed for similar optimization problems arising in image denoising [81, 84, 85], ICM is applicable to optimizing MRF at any dimension. The ICM procedure includes (i) initialization of the random variables (e.g. $\{\tilde{E}_{i,j}\}$ in 2D MRF) and (ii) selection of a single random variable to optimize in the loss function $\mathcal{L}$ while keeping all the other random variables fixed. Each round in (ii) requires computing at most five terms in the loss (Eq. (3) in the main text Methods) which depend on the selected random variable $\tilde{E}_{i,j}$. We can simply evaluate these terms at the energy axis values measured in the experiment to determine the energy associated with the lowest loss. (iii) iterate over all other random variables using the same procedure in (ii).

**2. Coding method**: The ICM procedure described above operates sequentially over every $\tilde{E}_{i,j}$, which is inefficient for the MAP optimization involving a large number of parameters. To improve the optimization performance, we implement the ICM with a checkerboard parallelization scheme (or coding method) [63] that scales favorably on multicore computing clusters. The scheme assigns the nodes of the MRF alternately with white and black colors, as shown in Supplementary Fig. 2a. If the white nodes are blocked, the black nodes are no longer connected through paths (i.e. sequences of connected edges and nodes). This property is called d-

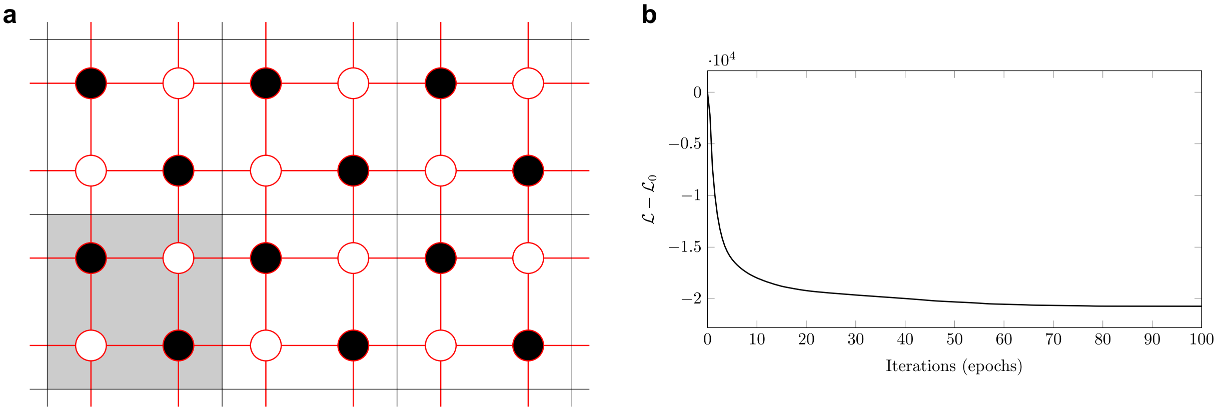


Supplementary Figure 2: **Numerical optimization of the MRF model**. **a**, Schematic of the checkerboard parallelization (or coding method) and hierarchical grouping schemes for speeding up the ICM. The nodes of the MRF are alternately colored white and black (checkerboard parallelization) and each set of four neighboring nodes are group into a unit as colored in grey (hierarchical grouping). The updates in optimization are carried out first at the four-node unit level, then alternately on the white or black nodes within the units. **b**, An example loss curve for reconstructing the second valence band of $WSe_2$ using the 2D MRF model and parallelized ICM implementation. $\mathcal{L}_0$ is the initial value of the loss at the start of the optimization. Within an epoch in the parallelized scheme, the white nodes and subsequently the black nodes are separately updated, therefore each band energy random variable is effectively updated once. The loss decreases rapidly in the beginning and reaches a minimum after about 90 epochs.

separation [81, 86]. Analogously, blocking the black nodes d-separates the white nodes. Since the MRF models satisfy the Hammersley-Clifford theorem [82], d-separation is equivalent to conditional independence, meaning that the random variables represented by the black nodes are independent if we condition on those represented by the white nodes. Therefore, conditioning on the nodes of one color allows us to compute the terms in the log-probability loss (Eq. (3) in main text Methods) that depends on the nodes of another color in parallel, which means that the nodes associated with different colors can be updated alternately. Further details and proofs related to the coding method have been elaborated in [83, 87].

**3. Hierarchical grouping**: The introduction of the checkerboard parallelization scheme reduces the translation symmetry of the original graph (originally symmetric by translation of an arbitrary number of nodes, now only symmetric by a translation of two nodes in each direction),

**Algorithm 1** Optimization procedure for reconstructing a single energy band.

**Input**: I (3D momentum-resolved photoemission data), $E_0$ (2D initialization from density functional theory calculation), E (1D energy axis)

**Parameter**: $\eta$ (hyperparameter of the Markov random field), N (number of epochs)

**Output**: $E_{rec}$ (Reconstructed 2D energy band)

```
# Initialize the momentum index grid for an energy band
size_kx, size_ky, size_E = size(I)
ind_x, ind_y = meshgrid(range(size_kx, step=2), range(size_ky, step=2))
# Divide data into four-node units. E_u(i,j,...), I_u(i,j,...) are the band energies and
# photoemission intensities for the node (i,j) in a unit (u) in Supplementary Fig. 2,
respectively
for i in [0, 1] do
    for j in [0, 1] do
        E_u[i, j, :, :] = E_0[ind_x + i, ind_y + j]
        log_I_u[i, j, :, :, :] = log(I[ind_x + i, ind_y + j, :])
# Iterative optimization of energy values
for n in range(N) do
    # Update white nodes
    E_u[0, 0, :, :] = update_E(0, 0, log_I_u, E_u, E)
    E_u[1, 1, :, :] = update_E(1, 1, log_I_u, E_u, E)
    # Update black nodes
    E_u[0, 1, :, :] = update_E(0, 1, log_I_u, E_u, E)
    E_u[1, 0, :, :] = update_E(1, 0, log_I_u, E_u, E)
# Assemble reconstruction from all nodes in the units
for i in [0, 1] do
    for j in [0, 1] do
        E_rec[ind_x + i, ind_y + j] = E_u[i, j, :, :]

# Function to update the energy of the element (i, j) within a four-node unit
function UPDATE_E(i, j, log_I_u, E_u, E)
    # Calculate the difference between current and all possible energies
    squ_diff = (E_u - E) ** 2 / (2 * η ** 2)
    # Calculate all possible log p values, start with log-likelihood
    log_p = log_I_u[i, j, :, :, :]
    # Substract by energy differences from nearest neighbor nodes within unit
    log_p -= squ_diff[(i + 1) % 2, j, :, :, :]
    log_p -= squ_diff[i, (j + 1) % 2, :, :, :]
    # Substract by energy differences from nearest neighbor nodes of the neighbor-
ing unit
    log_p -= shift(squ_diff[(i + 1) % 2, j, :, :, :], 2 * i - 1, axis=2)
    log_p -= shift(squ_diff[i, (j + 1) % 2, :, :, :], 2 * j - 1, axis=3)
    # Return optimal energy values
    return E[argmax(log_p)]
```

which complicates the matrix operations needed to update the loss. However, we can restore the translation symmetry and carry out the computation on a higher level by grouping a set of four neighboring nodes into a unit, as illustrated in Supplementary Fig. 2a. In this way, updating the loss requires only standard matrix operations at the unit level followed by consecutive updates of the nodes within the units. During the optimization, the loss is updated by two sets of operations concerning (i) the nearest neighbor nodes within the unit (line 18-19 in Algorithm 1) and (ii) the nearest neighbor nodes of the neighboring unit (line 20-21 in Algorithm 1). The latter operations are carried out by shifting the higher-level rectangular grid formed by the units by one step vertically or horizontally, followed by an operation on nodes of the respective units of the original and the shifted grid. The procedure is implemented in the open-source fuller package [64] using Tensorflow [62]. Supplementary Fig. 2b shows an example loss curve (i.e. loss as a function of iteration) in reconstruction of an energy band, where the optimization is essentially complete within $\sim$ 90 iterations.

**4. Robust initialization**: Since the current MRF model doesn't include any explicit regularization on the outcome with respect to the initialization, the optimizer is free to explore a large range of values. In other words, the initial band dispersion is able to freely deform to fit to the band loci embedded in the data. This design improves the robustness of the algorithm to initialization. As a result, in scenarios with only non-crossing energy bands, the MAP optimization can simply be initialized with constant energy values to yield consistent results. In general situations involving band crossings, the optimization procedure requires an initialization with approximate energy values that preserves the band-crossing information, such as those provided by electronic structure calculations. In this scenario, the robustness of the algorithm is manifest in the fact that it can tolerate a certain amount of deviation in the initialization and still converges to a satisfactory reconstruction, which, in realistic settings, is closer to the real band structure contained in photoemission data than the initialization (e.g. from electronic structure calculations). Quantitative examples demonstrating the robustness of initialization are provided

using synthetic data in Supplementary Figs. 6-9 (see Supplementary Section 2).

## 1.4 Hyperparameter tuning

The optimization process in the band structure reconstruction involves the tuning of three kinds of hyperparameters, which are the momentum scaling parameter, the rigid energy shift and the width of the nearest-neighbor Gaussian prior. A flowchart presented in Supplementary Fig. 3 illustrates the general steps in obtaining a desirable reconstruction including where the tuning of each hyperparameter fits in.

**1. Momentum scaling**: applied to equalize the momentum scale and resolution between the BS calculation (e.g. conducted on relaxed unit cells, see Supplementary Table 3) and the experimental data (measured on real materials). In our reconstruction procedure, the scaling factor is fixed in the reconstruction of all energy bands using a particular level of density functional theory (DFT) calculation as initialization.

**2. Rigid energy shift** (**$\Delta$E**): separately applied to each energy band in the calculated BS to coarse-align to the band mapping data. In our case, the shift is chosen manually by visual inspection of the theoretical band energies overplotted on photoemission data (usually in the energy-momentum slices). In practice, the necessary energy shifts vary between bands and also depend on the level of approximation in the BS calculation used as initialization, as illustrated in Fig. 2a of the main text.

**3. Width of the nearest-neighbor Gaussian prior** ($\eta$): The value of the parameter $\eta$ is chosen manually from an initial estimate and subsequently optimized by visual inspection of the reconstruction outcome. In the case of $WSe_2$, the momentum grid of the experimental data has a spacing of $\Delta k_x = \Delta k_y \approx 0.015$ Å$^{-1}$, we used $\eta \in [0.05, 0.2]$ eV. Generally speaking, the initial estimate of $\eta$ has the order of magnitude proportional to the momentum grid spacing times the dispersion due to the following argument: To obtain a consistent reconstruction, we expect the

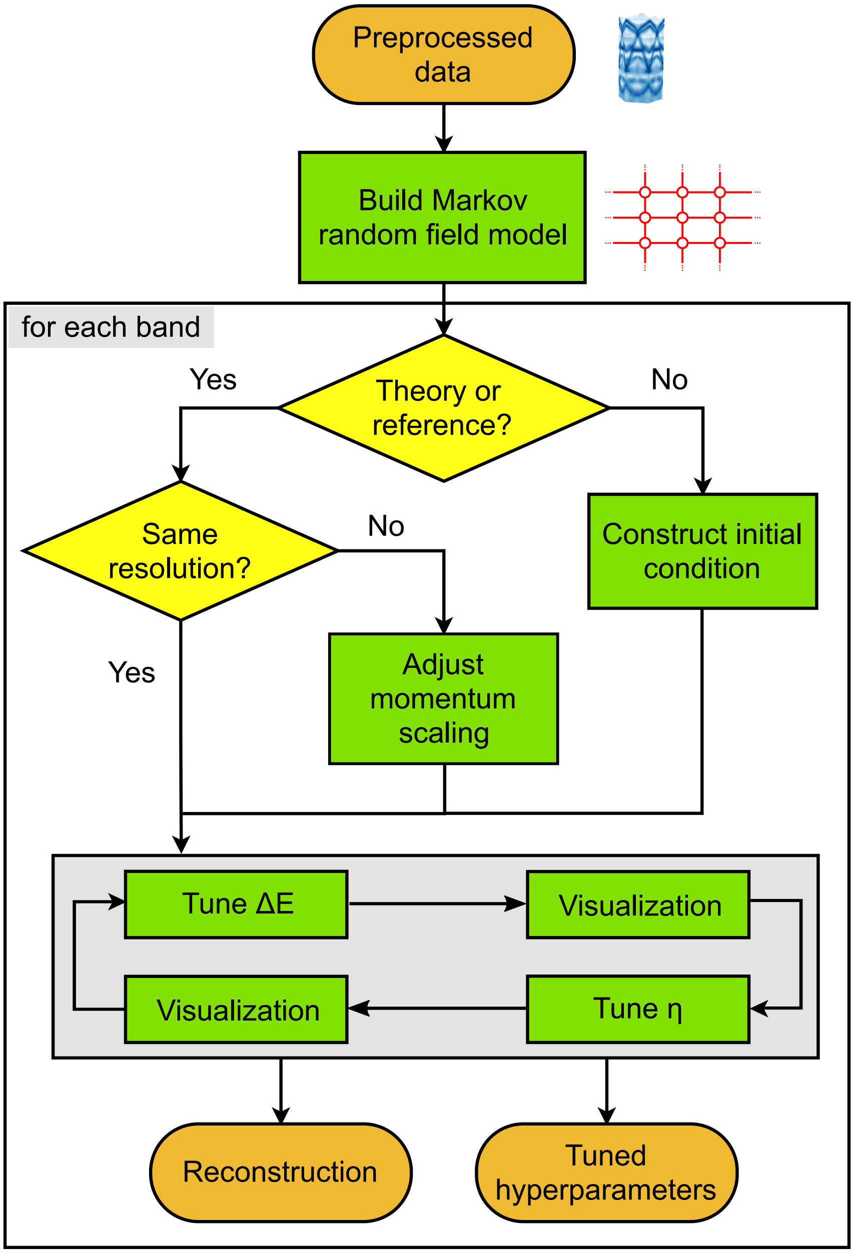


Supplementary Figure 3: **Flowchart for reconstruction tuning**. Illustration of the steps for tuning the reconstruction starting from preprocessed data (outcome from the procedure illustrated in the main text Fig. 1c-f). Tuning of the three hyperparameters – the momentum scaling, energy shift ($\Delta$E) and nearest-neighbor Gaussian width ($\eta$), are placed in sequence within the workflow. The workflow outputs reconstruction of a single band with tuned hyperparameters at the end. For reconstructing the dispersion of multiple energy bands, the workflow is repeated over each band.

posterior to stay relatively constant and be independent of the momentum grid spacing, which should be sufficiently fine to ensure band continuity. Since after preprocessing the data, the intensity (i.e. the likelihood) is normalized and stays constant with respect to the momentum grid spacing, the nearest-neighbor Gaussian prior term should stay constant correspondingly. For example, for two nearest-neighbor energy variables along the $k_x$ axis, the reasoning above requires,

$$\text{const} \approx \frac{(\tilde{E}_{i+1,j} - \tilde{E}_{i,j})^2}{\eta^2} \approx \left(\frac{\partial E}{\partial k_x}\right)^2 \frac{\Delta k_x^2}{\eta^2}. \tag{19}$$

Thereby, we obtain $\eta \propto \frac{\partial E}{\partial k_x}\Delta k_x$, which provide an order-of-magnitude estimate of $\eta$. The same lines of reasoning apply to the $k_y$ axis, for detector systems with relatively constant momentum resolution. As the grid spacing is the same in both $k_x$ and $k_y$ directions, a single $\eta$ is used for reconstructing each band in the case of $WSe_2$, but the best $\eta$ differs somewhat between energy bands due to their various amounts of dispersion and how they are connected to the neighboring bands (i.e. their environment), hence the range of $\eta$ as specified earlier.

To demonstrate the process of hyperparameter tuning, we provide an example showing the reconstruction of the second valence band of $WSe_2$ (see Supplementary Fig. 4), visualized in the top view of the reconstruction outcome and in the momentum path along high-symmetry lines of the projected Brillouin zone. The orange-framed subfigures represent the range of hyperparameter settings that yield a good reconstruction, which represents a relatively broad acceptance range to yield a good reconstruction. Although this aspect is dependent on the data, in our experience, the hyperparameter tuning may be carried out in a semi-automated fashion guided by visualization and heuristics. Typically, 10-20 trials are sufficient to yield a good reconstruction, although a grid search may also be carried out for completeness. For a given dataset, the hyperparameters typically fall within a similar range, therefore, determining the range of hyperparameters need only be carried out once. The choice of hyperparameters is more flexible for reconstructing more isolated bands or those with fewer crossings, and *vice*

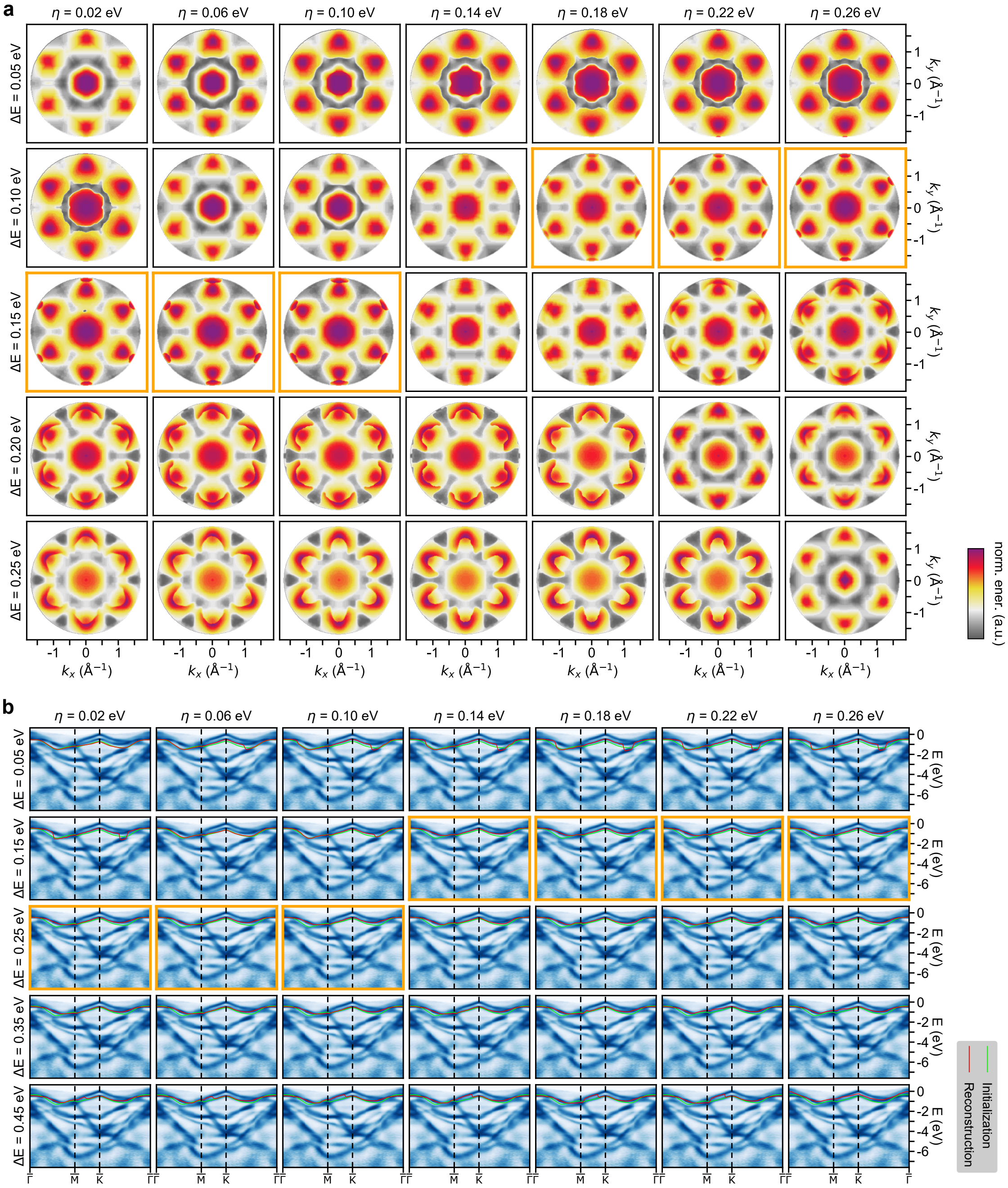


Supplementary Figure 4: **Demonstration of hyperparameter tuning**. An example of tuning the hyperparameters, the rigid energy shift (ΔE) and the width of the nearest-neighbor Gaussian prior ($\eta$), for reconstructing the second valence band of $WSe_2$. **a**, Evolution of reconstructed energy band during hyperparameter tuning. **b**, Evolution of the initialization and reconstructed band along high-symmetry directions of the hexagonal lattice of $WSe_2$. The energy bands are overlaid on top of preprocessed data from photoemission band mapping of $WSe_2$ (Fig. 1f in the main text). In **a**,**b**, the images showing the optimal region for the hyperparameters identified by the scientists are emphasized with orange-colored frames.

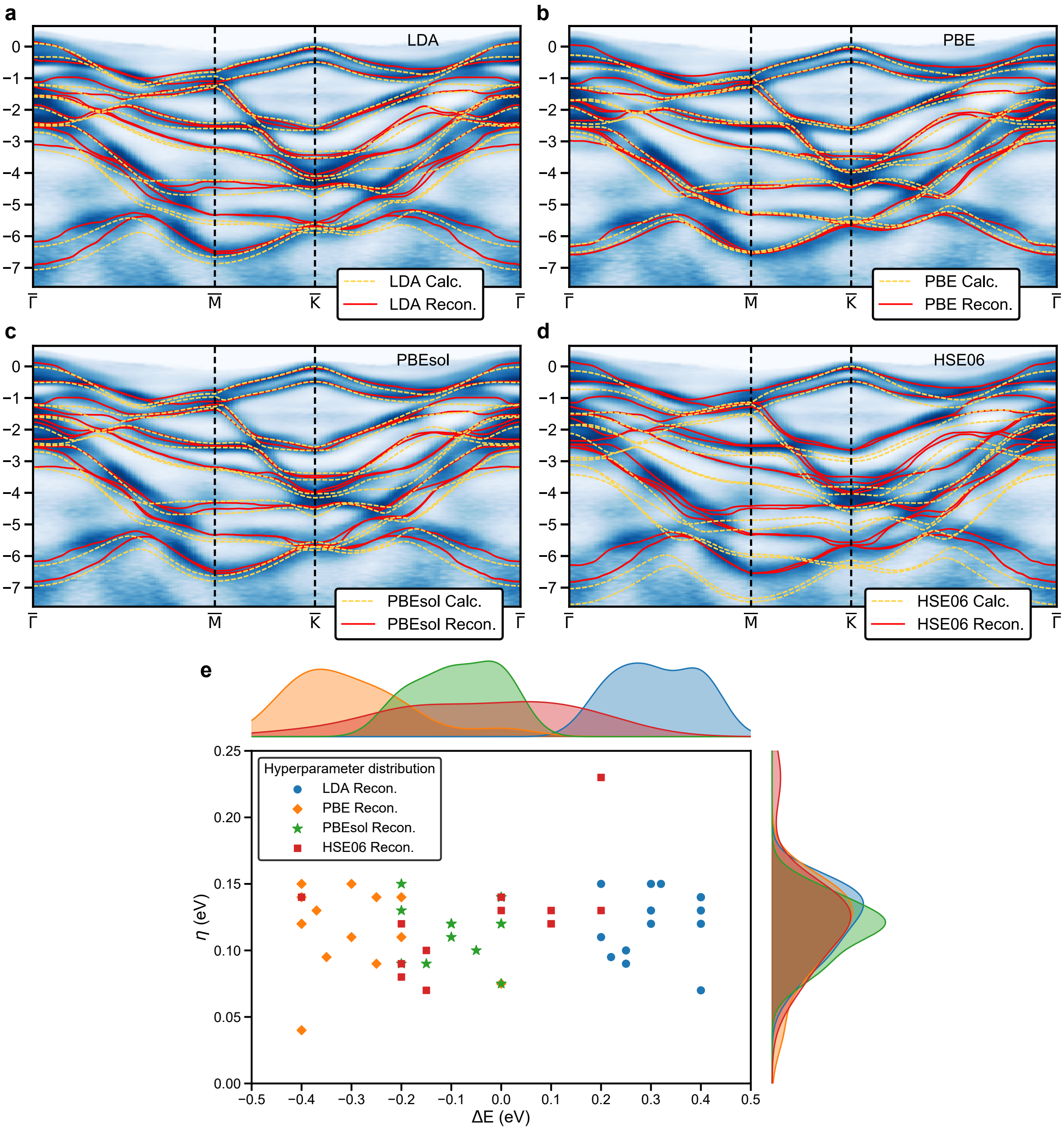


Supplementary Figure 5: **Band structure reconstructions with different theory initializations**. Comparisons between reconstructed photoemission band structures (abbreviated as recon.) and calculated band structures (abbreviated as calc.) from density functional theory (DFT) with different exchange-correlation functionals, including **a,** local density approximation (LDA); **b,** PBE generalized gradient approximation (GGA); **c,** PBEsol GGA; **d,** HSE06 hybrid functional. For each set of DFT band structure, the same energy shift (as in Supplementary Fig. 12) is applied globally to all bands to align the energy zero at the $\overline{\mathrm{K}}$ point with the reconstruction. **e,** The distribution of hyperparameters used for the reconstruction in **a-d**.

*versa*. The band-wise reconstruction and the computational efficiency of the algorithm also enable further parallelization in hyperparameter tuning by distributing the optimization tasks in a high-performance computing infrastructure.

### 1.5 Reconstructions using different theories as initializations

Comparison between reconstructed and theoretical band structures for $2H$-$WSe_2$ are presented as a similarity matrix in the main text. To provide more intuitive visual guidance in interpreting the BS distance metric used in constructing the similarity matrix, we compare these band structures along the high-symmetry lines of the Brillouin zone in Supplementary Fig. 5.

Here, the comparison between reconstructed and calculated band structures show that the HSE06 and PBE have, respectively, the largest and smallest overestimation of total band width of $WSe_2$ among the four initializations, though HSE06 has a higher level of chemical accuracy than PBE [30]. The calculated band structures are closer to the reconstruction near the $\overline{\mathrm{K}}$-point than elsewhere in the projected Brillouin zone, reflecting the difference in electronic dimensionality between $\overline{\mathrm{K}}$ (nearly ideally 2D) and elsewhere [11].

## 2 Generation of and validation on synthetic data

The advantage of using synthetic data is that the underlying band structure (i.e. ground truth) is exactly known so it can be used for benchmarking the performance of the MAP reconstruction algorithm described in this work. Benchmarking includes numerical experiments on two interrelated aspects: (1) testing the robustness of the reconstruction algorithm using different initializations and comparing the deviations of the outcome from the ground-truth; (2) testing the accuracy of reconstruction by determining the closest-possible reconstruction outcome from a given initialization. In the following, we first describe the workflow of generating the band structure, the photoemission data and the initializations, which provide all essential components

to carry out the tests. Then we present the benchmarking results on various cases.

## 2.1 Generation of band structure data

We have adopted two approaches to generate band structure data to meet the needs for testing the reconstruction algorithm. Firstly, we used analytic functions to describe the band dispersion (see Supplementary Fig. 6). They are computationally efficient, contain tunable parameters, can be produced at any resolution, and are easily extendable to higher dimensions. In 2D momentum space, we constructed a multi-sinusoidal band and two double-crossing parabolic bands. In 3D momentum space, we constructed a scaled version of the strongly oscillating second-order Griewank function [88] and the tight-binding formulation of the two-band graphene band structure [89] as model band dispersion surfaces. The modified Griewank function takes the form,

$$E_{\text{griewank}}(k_x, k_y) = \frac{1}{16000}(k_x^2 + k_y^2) - \cos(2k_x)\cos(\sqrt{2}k_y). \tag{20}$$

The two-band tight-binding model of graphene has energy dispersion relations,

$$E_{\pm}(k_x, k_y) = \pm\sqrt{3 + 2\cos\left(\sqrt{3}k_y a\right) + 4\cos\left(\frac{\sqrt{3}}{2}k_y a\right)\cos\left(\frac{3}{2}k_x a\right)}. \tag{21}$$

Here, $E_+$ and $E_-$ refer to the conduction band and the valence band, respectively. Secondly, we used numerical band structures from DFT calculations with different exchange-correlation functionals (see Supplementary Section 4). They are more physically realistic, but also require more computation to obtain than generating bands from analytic functions.

## 2.2 Initialization tuning

For simple bands constructed using analytic functions, tuning can be achieved by modifying the parameters in the functions. In complex multiband situations such as that of $WSe_2$, we

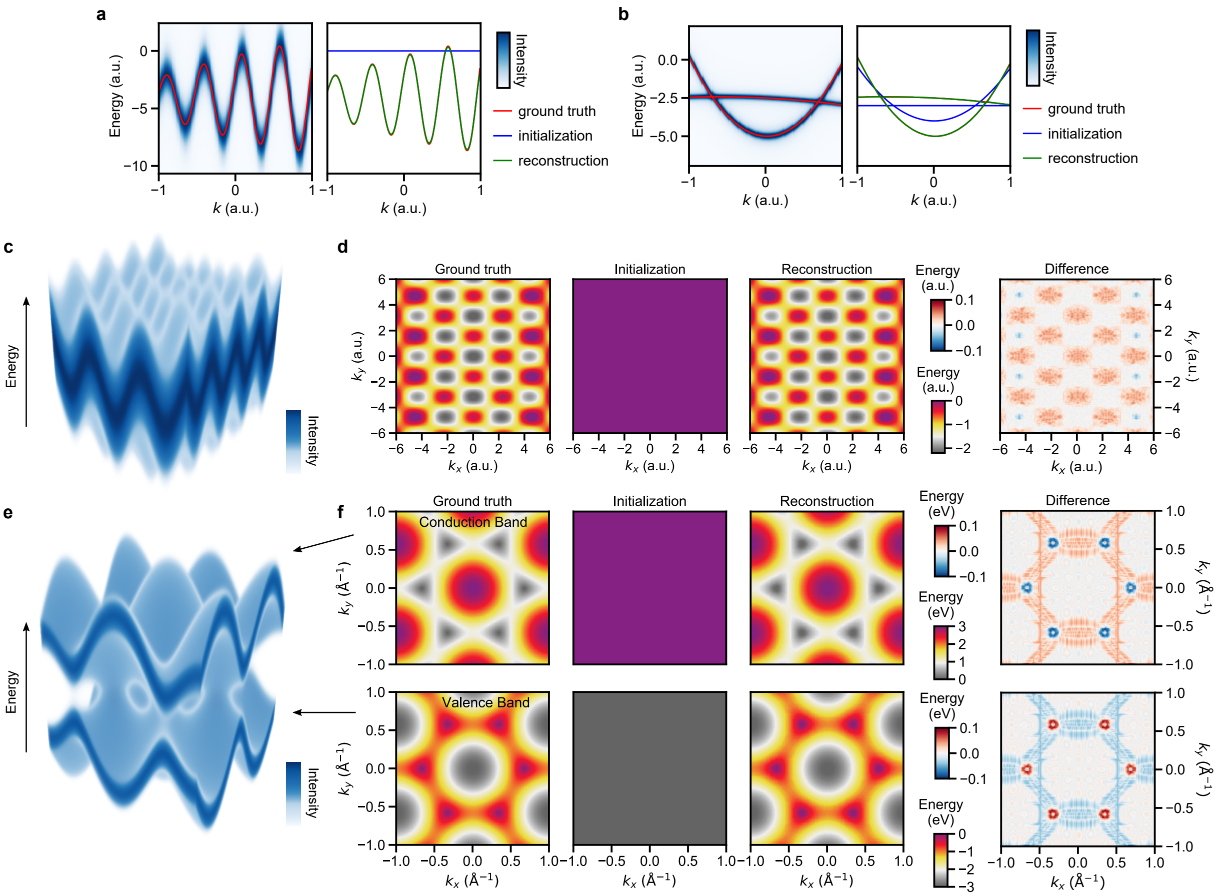


Supplementary Figure 6: **Validations on 2D and 3D synthetic data**. Test results for the reconstruction algorithm on band structures generated with analytic functions. **a**, Reconstruction of a multi-sinusoidal band. **b**, Reconstruction of two double-crossing parabolic bands. **c**,**d**, Reconstruction of a multi-extrema band with dispersion following the second-order Griewank function (see Eq. (20)) [88]. **e**,**f**, Reconstruction of the two bands of graphene nearby its Fermi level (**e**,**f**) formulated in the tight-binding model (see Eq. (21)) [89]. The volumetric renderings in **c**,**e**, display the synthetic data. The initialization for the reconstruction in **a** is a flat line, while 2D flat bands are used to initialize the cases in **d**,**f**. In **b**, two double-crossing curves are needed as initialization to preserve the crossing in the reconstruction. The values in the difference plots in **d**,**f** are calculated by subtracting the ground-truth band energies from the reconstructed ones.

tuned the initialization of the reconstruction algorithm by scaling or perturbing the coefficient amplitudes of the constituent bases of the band structure. In our case, the bases are the terms of the hexagonal Zernike polynomials (ZPs) [35, 90]. Although unconstrained basis tuning is prone to unrealistic results, it achieves a level of ad hoc control for the efficient generation of a large amount of distinct initializations. For more physically realistic tuning, we used DFT

calculations with different exchange-correlation functionals (see Supplementary Section 4).

## 2.3 Approximate generation of photoemission data

We approximately synthesized momentum-resolved photoemission data for each energy band by plugging the band energy and linewidth parameter at each momentum position into the Voigt profile [91] (with Gaussian and Lorentzian parameters $\sigma$ and $\gamma$, and amplitude $B$) computed using the Faddeeva function $W$ [92]. The Voigt profile approximates the convolution of a single-particle spectral function (see Supplementary Section 1), describing the photoemission observable, with a Gaussian energy resolution function. The synthetic photoemission intensity, $I_{\text{synth}}$, for a band structure composed of a set of energy bands, $E_B = \{E_{b_i}\}$, is generated by combining multiple Voigt profiles in summation, similar to Eqs. (13)-(14).

$$I_{\text{synth}}(k_x, k_y, E) = \sum_j \frac{B_j(k_x, k_y)}{\sigma_j\sqrt{2\pi}} \text{Re}\left[W\left(\frac{E - E_{b_j}(k_x, k_y) + \mathrm{i}\gamma_j(k_x, k_y)}{\sigma_j\sqrt{2}}\right)\right] \tag{22}$$

Without loss of generality, we assume the energy resolution in detection for all bands to be the same ($\sigma_j = \sigma$). For the cases shown in Supplementary Figs. 6-9, the linewidth parameter $\gamma$ are set to a constant throughout the band. In all synthetic data, we omitted the inhomogeneous intensity modifications in realistic photoemission data due to experimental factors such as the experimental geometry, sample condition, matrix element effect, photon energy, etc. This omittance relies on the assumption that the essential preprocessing step, such as symmetrization and contrast enhancement [29] in our workflow (see main text Methods), can sufficiently restore the intensity continuity along the energy bands. The momentum resolution effect is also not accounted for because the instrument (such as METIS 1000 [8, 59]) has a higher momentum resolution than the momentum spacing used in data binning or generation.

## 2.4 Validation of the reconstruction algorithm

Using synthetic data generated from analytic functions of varying complexities as the band structure, we test out the accuracy of the reconstruction algorithm (see Supplementary Fig. 6); Using synthetic multiband data generated from the LDA-level DFT (LDA-DFT) band structures of $WSe_2$ (see Supplementary Section 4), we tested out the sensitivity of reconstruction to the initialization (see Supplementary Fig. 9). In this case, to capture sufficient physical realism similar to the photoemission band mapping of $WSe_2$ presented in the main text, we set the energy resolution parameter of $\sigma$ = 100 meV, the lineshape parameter $\gamma$ = 50 meV [93], and the energy spacing of data to $\sim$ 18 meV, identical to the energy bin size for the experimental data. The tests include four sets of numerical experiments summarized below:

**1. Reconstructing non-crossing bands**: For isolated bands, we tested synthetic data constructed from a multi-sinusoidal band (Supplementary Fig. 6a), the band generated by the Griewank function (Supplementary Fig. 6c-d), and the two-band tight-binding model of graphene (Supplementary Fig. 6e-f). In these cases, initialization with a flat band without any initial knowledge of the band dispersion (i.e. cold start) is sufficient to recover its shape, regardless of the complexity of the dispersion.

**2. Reconstructing crossing bands**: We tested the simplest case of crossing bands with two parabolas of opposite directions of opening (Supplementary Fig. 6b), a recurring pattern in band structures. To recover the dispersion without band index scrambling, the knowledge of crossing needs to be included numerically in the initialization. This means, operationally, that the initialization requires crossing bands at nearby energy values, or that the reconstruction needs a warm-start optimization. For the double-crossing parabolas, the initializations that yield feasible outcomes are generated by slight tuning of the parabola parameters in the range that retains the crossing. A careful examination over possible scenarios largely confirms this intuition: (1) Initialization with parallel straight lines (without any crossing) only results in non-

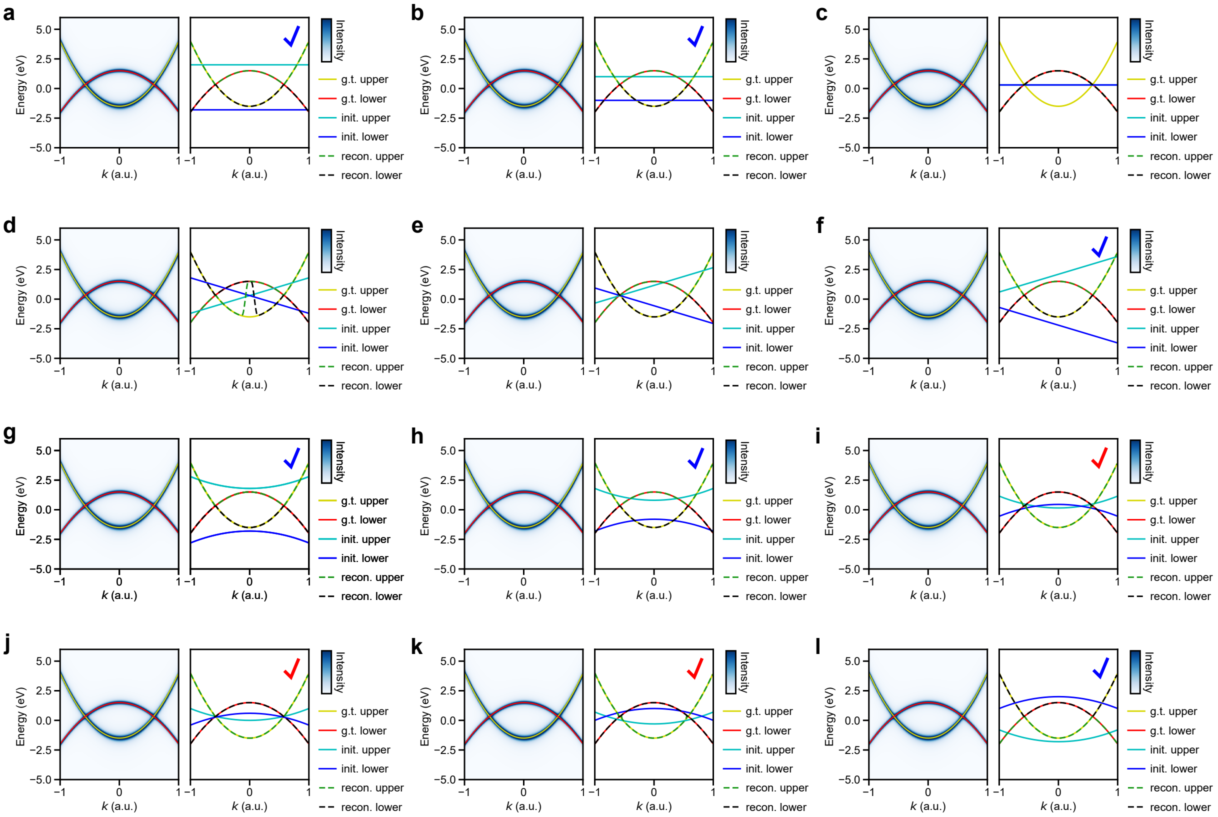


Supplementary Figure 7: **Essential information in initialization for reconstruction**. Results from a series of numerical experiments for demonstrating the effects of band-crossing information in the initialization. For clarity, the results are compared against the ground truth (g.t.) band dispersion – double-crossing parabolas – by overplotting in dashed lines. The tuning involves initializing the reconstruction with three sets of common curves: **a**-**c**, parallel straight lines, **d**-**f**, single-crossing straight lines, and **g**-**l**, double parabolas. The red check marks (✓) label the reconstructions with correct crossings, while the blue check marks (✓) label those with anti-crossings. All numerical experiments used the same simulated data from a toy model with double-crossing parabolas containing only the second and zeroth-order terms. For reconstruction experiments, the nearest-neighbor Gaussian width hyperparameter ($\eta$) in the MRF model is tuned, while the relative position of the initial conditions is shifted to each configuration.

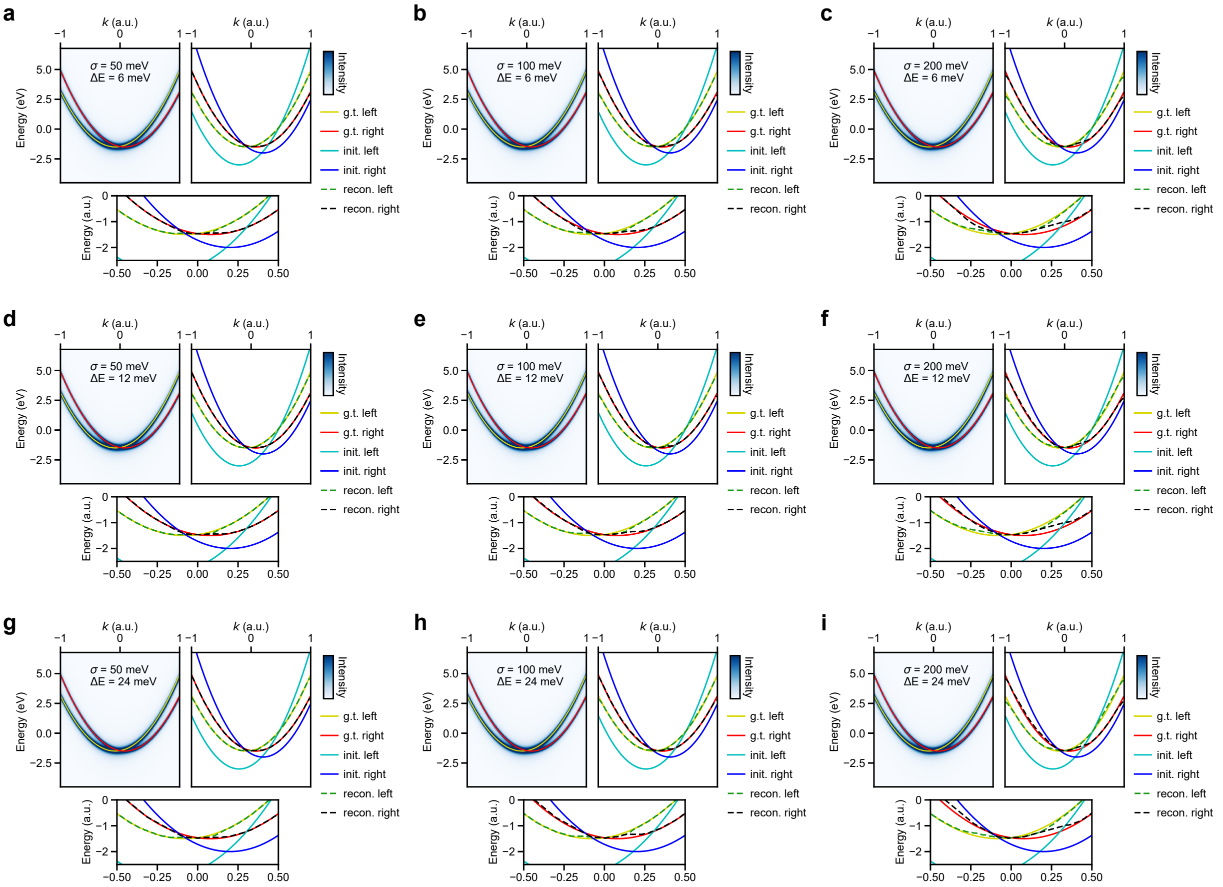


Supplementary Figure 8: **The effects of data resolution on reconstruction**. Results from a series of numerical experiments for demonstrating the effects of data resolution in either instrument resolution ($\sigma$) or energy spacing of data ($\Delta E$) on the reconstruction accuracy. The results are compared against the ground truth (g.t.) band dispersion – displaced parabolas – by overplotting in dashes lines. The $\sigma$ parameter is tuned to 50 meV, 100 meV and 200 meV, while the $\Delta E$ parameter to 6 meV, 12 meV and 24 meV. In **a-i**, the synthetic data with ground truth dispersion is shown on the left, the reconstruction outcome is displayed on the right, along with a zoomed-in view near the crossing placed at the bottom. Quantitative values of the reconstruction error are given in Supplementary Table 1.

crossing bands in the reconstruction (Supplementary Fig. 7a-c). When the initial straight line contains the crossings in the ground truth, a symmetry breaking in the reconstruction takes place (Supplementary Fig. 7c), depending on the data and the Gaussian width hyperparameter ($\eta$). (2) Initialization with two straight lines containing a single crossing yields a reconstruction with at most a single crossing (Supplementary Fig. 7d-f). (3) Initialization with double parabolas yields a reconstruction with at most the same number of crossings within the range of the data (Supplementary Fig. 7g-l). When the reconstruction is successful, the crossings in the initialization are close to the intersection between the two parabolas. Besides, double-crossing parabolas with other parameters from those in Supplementary Fig. 7 are tested and similar outcomes are obtained.

Supplementary Table 1: **Reconstruction error in resolution tuning experiments.** For each band, the reconstruction error is the root-mean-square error per momentum spacing (unit in meV) between reconstruction and the ground truth, according to Eq. (9). In each numerical experiment, the tabulated reconstruction error is averaged over the corresponding two parabolic bands shown in Supplementary Fig. 8. The columns are the instrument resolution ($\sigma$) and the rows are the energy spacing ($\Delta E$) used to generate the intensity data.

| | $\sigma$ = 50 meV | $\sigma$ = 100 meV | $\sigma$ = 200 meV |
|---|---|---|---|
| $\Delta E$ = 6 meV | 2.1 | 3.7 | 7.5 |
| $\Delta E$ = 12 meV | 2.3 | 3.8 | 7.9 |
| $\Delta E$ = 24 meV | 2.4 | 4.6 | 10.4 |

The crossing-band model is also an effective test case for resolution effects of the reconstruction algorithm. In this case, a momentum shift is introduced to two parabolic bands to produce the crossing, similar to the Rashba-split surface states of Au [94], which if often used to calibrate experimental resolution in photoemission studies. We conduct a series of numerical experiments using different widths of the instrument resolution and energy spacing to simulate the resolution effect in the synthetic data, using reasonable parameter values. All numerical experiments use a nearest-neighbor Gaussian width hyperparameter $\eta$ within $[0.08, 0.11]$ for the reconstruction and no rigid energy shift is introduced. We tabulate the outcomes visually in

Supplementary Fig. 8 as gridded figures and quantitatively in Supplementary Table 1 using the reconstruction error (root-mean-square error between ground truth and reconstruction) with the unit in meV. These results show that the reconstruction accuracy, as quantified by the error, has the same trend as the data resolution, which is determined by both the instrument resolution and energy sampling. The instrument resolution appears to have a larger effect on the reconstruction than the energy spacing. In other words, the worse the data resolution ($\sigma$ = 200 meV and $\Delta E$ = 24 meV being the worst case), the higher the reconstruction error. From visual inspection of the reconstruction in Supplementary Fig. 8, including the zoomed-in region where the crossing is present, it appears that these changes in reconstruction accuracy create essentially no difference in the band dispersion away from the band crossing and only a marginal difference in the vicinity of the band crossing.

**3. Sensitivity of reconstruction to scaled energies as initialization**: We scaled the energies of the LDA-DFT band structure of $WSe_2$ (using the first 8 valence bands) around the mean energy of each band (see Supplementary Fig. 9c) for use as the initialization. The accuracy of the reconstruction outcome is evaluated by its average error $\eta_{\rm avg}$ (Eq. (8) in the main text Methods), calculated with respect to the ground-truth band energies. The results displayed in Supplementary Fig. 9d,f show that the average error and its spread in the reconstruction are reduced from the corresponding values in the initialization. Quantitatively, in the reconstruction, $\eta_{\rm avg}$ is within the range 20-65 meV, while in the initialization, $\eta_{\rm avg}$ varies within 45-100 meV for all 8 valence bands.

**4. Sensitivity of reconstruction to differently calculated band structures as initialization**: We used DFT band structure calculations of $WSe_2$ with PBE, PBEsol and HSE06 exchange-correlation functionals (see Supplementary Section 4) to initialize the reconstruction. The accuracy of the reconstruction is quantified similarly as in the previous numerical experiment using $\eta_{\rm avg}$. The results displayed in Supplementary Fig. 9e,g,h show that, despite the huge spread

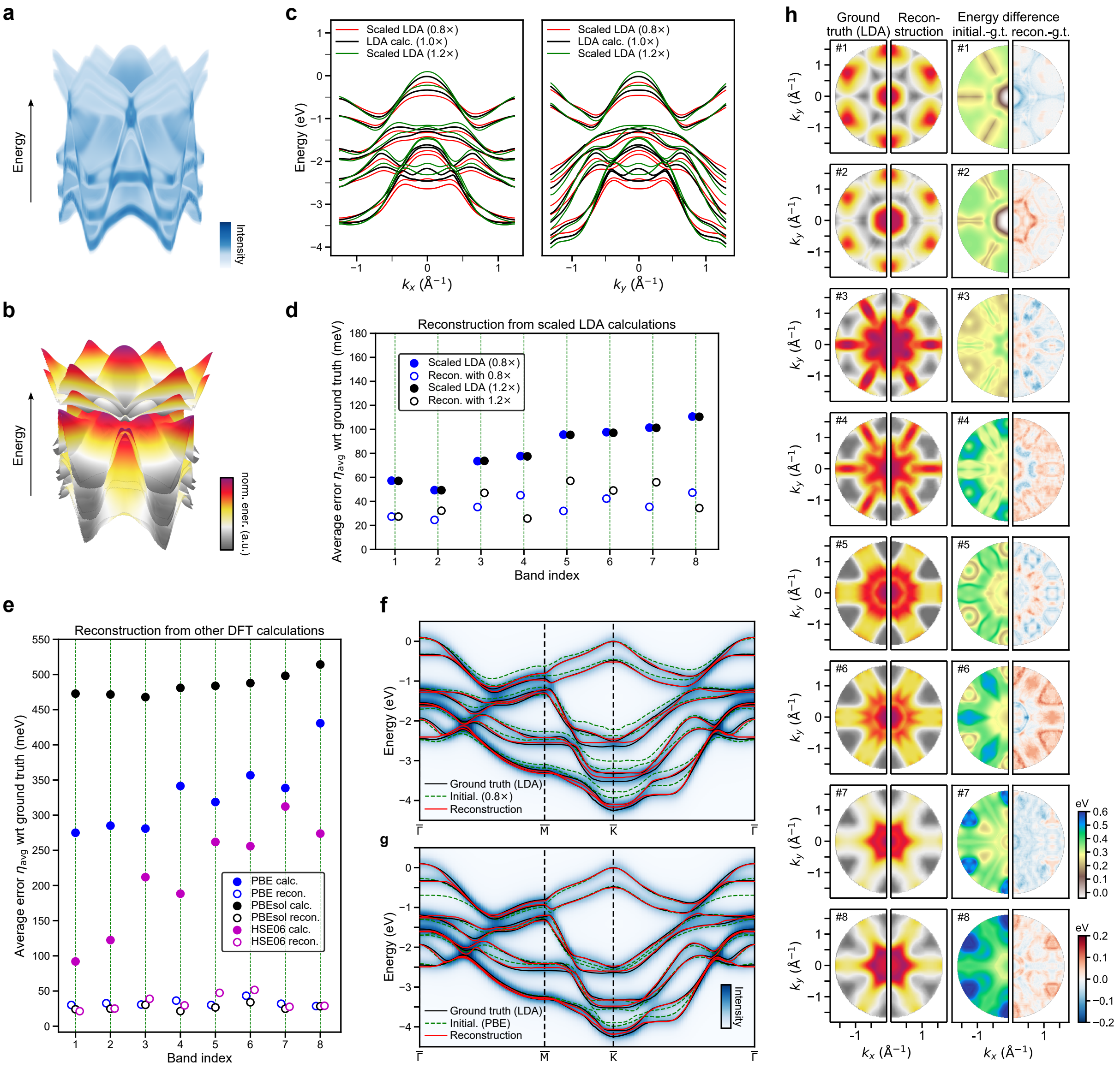


Supplementary Figure 9: **Validation on 3D synthetic multiband photoemission data**. **a**, Synthetic photoemission data with **b**, the underlying band structure obtained from LDA-level DFT calculation of $WSe_2$ (only the first 8 valence bands are used here). **c**, Comparison of two sets of differently scaled (by 0.8 and 1.2 times, respectively) initial conditions with respect to the ground-truth band structure calculation (LDA calc.), shown for a $k_x$-$E$ (left) and a $k_y$-$E$ (right) slice. **d**,**e**, Comparison of the average error $\eta_{\text{avg}}$ for energy bands used as initializations (solid dots) and reconstructions (hollow dots). The initializations are constructed by scaling the ground-truth band energies (**d**) or by using other DFT calculations (**e**). The reconstructions all have reduced $\eta_{\text{avg}}$ compared with the initialization and $\eta_{\text{avg}}$ is consistent across all energy bands. **f**,**g**, Reconstruction, ground truth (LDA), and initialization overlaid on the synthetic data along high-symmetry lines of the hexagonal Brillouin zone, corresponding to two of the cases in **d** and **e**, respectively. The energy zeros of the initialization in **d**-**e** are aligned with the ground truth via a global shift. **h**, Comparisons of ground truth (LDA), reconstructed bands, and the differences between initialization (PBE), reconstruction and ground truth (g.t.) for each band.

in the average error for the different levels of DFT calculations (used as initialization without global shift alignment of energy zero), the corresponding reconstructions all have average errors at around or below 40 meV for every band. The value of $\eta_{\text{avg}}$ varies by up to $\sim$ 30 meV (i.e. between band #1 and #6) in each set of reconstructed bands, much lower than those in the initialization. The former can be improved by casting the experimental data into finer bins in the preprocessing stage (single-electron events can be binned into various sizes) or interpolating between existing bins, while the latter can be improved by using a continuous probabilistic model [81] to formulate the reconstruction problem, albeit at the cost of much increased computational demand.

The results of the above numerical experiments demonstrate that the reconstruction by MAP optimization converges to a consistent range in the tested scenarios and initializations. It should be noted here that the fundamental accuracy in reconstruction reported here is still limited by the coordinate spacings of the data along all dimensions and the discrete nature of the MRF model (the output is centered only at the bin locations).

## 2.5 Computational benchmarks

We used the available synthetic photoemission datasets based on the computed band structure of $WSe_2$ to construct benchmarks. The synthesis made use of the approach described in Supplementary Section 2.1. The two datasets used here, taken from [95], exhibit different characteristics, which may be qualitatively described using the energy range of an energy band. The more overlap in the energy range between two energy bands, the more likely they have crossings (or anti-crossings). The dataset-specific information is as follows:

- The synthetic dataset of the $WSe_2$ K-point shows close proximity in energies between neighboring momentum locations. The energy ranges of all energy bands have no or up to a moderate degree of overlap. The dataset size is $30 \times 30 \times 500$ and contains 900 photoemission spectra.

- The synthetic high-symmetry line dataset of $WSe_2$ exhibits large dispersion. Since the high-symmetry line often represents the direction with the most dispersion in the band structure, the energy ranges of all energy bands are strongly overlapping. The dataset size is 186 $\times$ 500 and contains 186 photoemission spectra.

In both cases, the ground-truth band dispersions are taken from the LDA-DFT calculation, including all 14 valence bands, while the initializations for benchmarking both band reconstruction approaches are the PBE-DFT calculation (partial example see Supplementary Fig. 9g). Using these two datasets, we compare the reconstruction algorithms based on pointwise fitting (using the code in [40]) and MRF as introduced in this work. The hyperparameters for the pointwise fitting involve only the band-wise relative shifts applied in each band initialization (14 hyperparameters in total for 14 bands), which were tuned for each band sequentially from band #1 to band #14 using an expanding window approach (introduced in [40]). For the MRF reconstruction, the hyperparameters (including the band-wise shift and the width of the nearest-neighbor Gaussian prior, 28 hyperparameters in total for 14 bands) were tuned individually while reconstructing each band. The hyperparameter tuning made use of grid search through a range of preset values, using the root-mean-squared (RMS) error for determining the final choice. All benchmarks were run on an on-premises computing server (Dell PowerEdge R840), equipped with four Intel Xeon Gold 6150 multicore CPUs.

The computational performance of the two algorithms was evaluated using four different metrics as summarized in Supplementary Table 2. The timing metrics provided in the table include the average single-run computing time in each dataset as well as the total computing time of the hyperparameter tuning, which covers all grid search steps of the energy bands (indices described in $R_{\mathrm{band}}$) in every benchmarking stage. The computing time for the single runs of each dataset shows a clear advantage of the machine learning-based algorithm and the gap between the two algorithms only widens as the number of bands increases. The accuracy of the reconstruction is quantified by an RMS error averaged over all reconstructed bands and spectra,

Supplementary Table 2: **Algorithm comparison using benchmark datasets.** Two synthetic datasets with different number of spectra ($N_{\text{spec}}$) and the range of band indices ($R_{\text{band}}$) are used for benchmarking the algorithm performance. The per-band, per-spectrum reconstruction error ($\eta_{\text{bk}}$) is calculated using Eq. (23). The instability ($r_{\text{band}}$) quantifies the variation of the fitting residuals among all spectra within a dataset using the standard deviation of residuals, as in Eq. (24). The single-run time ($t_{\text{mono}}$) is the averaged elapsed time in a single execution of fitting, while the tuning time ($t_{\text{tune}}$) is the total time used for tuning the parameters to reach the final outcome. Both methods use DFT calculation as the initialization for the band positions.

| Dataset [1] | $N_{\text{spec}}$ | $R_{\text{band}}$ | Pointwise line fitting [2] | | | | MRF reconstruction | | | |
|---|---|---|---|---|---|---|---|---|---|---|
| | | | $t_{\text{mono}}$ (s) | $t_{\text{tune}}$ (s) | $\eta_{\text{bk}}$ (eV) | $r_{\text{band}}$ (eV) | $t_{\text{mono}}$ (s) | $t_{\text{tune}}$ (s) | $\eta_{\text{bk}}$ (eV) | $r_{\text{band}}$ (eV) |
| $WSe_2$ K point | 900 | 1-2 | 42 | 421 | **4.6e-4** | **3.2e-4** | **6.6e-1** | **34** | 5.8e-3 | 4.1e-3 |
| | | 3-4 | 168 | 2519 | 3.6e-1 | 1.4e-1 | **1.0** | **96** | **1.1e-2** | **5.5e-3** |
| | | 5-8 | 412 | 11964 | 1.5e-1 | 5.3e-2 | **2.2** | **134** | **6.0e-2** | **2.1e-2** |
| | | 9-14 | 2792 | 78181 | 3.6e-1 | 9.6e-2 | **3.8** | **236** | **7.8e-2** | **2.1e-2** |
| $WSe_2$ high-symmetry line | 186 | 1-2 | 13 | 191 | 3.6e-1 | 2.1e-1 | **3.9e-1** | **32** | **1.1e-2** | **2.0e-2** |
| | | 3-4 | 46 | 692 | 6.2e-1 | 2.9e-1 | **2.9e-1** | **31** | **1.9e-2** | **1.8e-2** |
| | | 5-8 | 385 | 8858 | 5.5e-1 | 1.7e-1 | **8.6e-1** | **56** | **3.0e-2** | **1.4e-2** |
| | | 9-14 | 872 | 27889 | 3.3 | 8.7e-1 | **1.6** | **109** | **4.1e-2** | **1.1e-2** |

[1] Datasets are obtained from [95].
[2] Executed using the software described in [40].

following the expression for "band delta" in [72].

$$\eta_{\text{bk}}(E_{\text{gt}}, E_{\text{recon}}) = \sqrt{\frac{1}{N_b N_{\text{spec}}} \sum_{i=1}^{N_b} \sum_{\mathbf{k}} (E_{\text{gt},i,\mathbf{k}} - E_{\text{recon},i,\mathbf{k}})^2}, \tag{23}$$

where $N_b$ is the number of bands and the subscript $i$ is the band index. The instability is quantified by the standard deviation of the residual (difference between the ground truth and reconstructed energy dispersion), $\delta E = E_{\text{gt}} - E_{\text{recon}}$.

$$r_{\text{band}}(E_{\text{gt}}, E_{\text{recon}}) = \sqrt{\frac{1}{N_b} \sum_{i=1}^{N_b} \sum_{\mathbf{k}} (\overline{\delta E_{i,\mathbf{k}}^2} - \overline{\delta E_{i,\mathbf{k}}}^2)}, \tag{24}$$

where the overline indicates the mean. This metric has been used in earth sciences to quantify surface roughness [41, 96], which may be interpreted similarly in our case. The difference is that the roughness in the reconstructed surface is largely due to the instability of the optimization, besides the quality of the data, because band dispersions are generally smooth and continuous. In the main text Fig. 4, these tabulated metrics are normalized by the number of spectra to allow comparison between datasets, as is also adopted in [40]. We interpret the results in Supplementary Table 2 in the following two aspects:

- Computing time ($t_{\rm mono}$ and $t_{\rm tune}$): For the same dataset, the single-run computing time of the MRF reconstruction is about 2-3 orders of magnitude faster than distributed pointwise fitting. Even with the hyperparameter tuning included, the MRF reconstruction still runs 1-2 orders of magnitude faster, although the MRF reconstruction requires tuning one more hyperparameter than the pointwise fitting approach for each band.

- Reconstruction quality ($\eta_{\rm band}$ and $r_{\rm band}$): The substantially higher reconstruction error and instability for pointwise fitting are due to the lack of connectivity between neighbors come primarily from the (theoretical) initialization. Because for each band, even though a global energy shift hyperparameter is tuned, it cannot guarantee that everywhere locally the shift is optimal for band reconstruction, resulting in scrambled band indices in the local patches that the fitting fails. This scenario is a failure mode of the pointwise fitting-based reconstruction as illustrated in [40] for real-world experimental data. This limitation of the pointwise fitting approach is less pronounced when the energy range overlap between bands is small, yet becomes more severe in the high-symmetry line dataset, where the strong energy range overlap and the multiple band crossing (or anti-crossing) make the reconstruction harder to resolve by tuning a single energy shift parameter. The probabilistic framework of the MRF approach largely circumvents this limitation using a physical prior that accounts for the proximity of the neighboring energy values and achieves high stability in the reconstruction.

# 3 Reconstruction for other datasets

To test the functionality of our MRF reconstruction algorithm on other materials, we have acquired photoemission band mapping datasets from gold (Au), a metal, and bismuth tellurium selenide ($Bi_2Te_2Se$), a topological insulator. Due to the complexity of the electronic structure of these materials, we focus here on reconstructing a subset of the energy bands of these two materials that are pronounced within the measured energy range. Besides, we simulated the case where the electron self-energy strongly modifies the band dispersion that results in kink anomalies [6, 97, 98].

## 3.1 Near-gap electronic bands of a topological insulator ($Bi_2Te_2Se$)

The dataset for $Bi_2Te_2Se$ was measured at room temperature at the Fritz Haber Institute in Berlin using a momentum microscope (SPECS METIS 1000). The sample growth method was previously described in [99]. A clean surface was prepared in vacuum by *in situ* cleaving with Scotch tape. During the measurement, light excitation of 800 nm was used to examine ultrafast dynamics. The temporal features were ignored here and averaged to improve the signal-to-noise ratio of the data. The photoemission spectra of $Bi_2Te_2Se$ near the Fermi level feature a topologically-protected surface state (SS) that intersects at the Dirac point (DP) [43] as shown in main text Fig. 6a-b. The SS bridges the valence and conduction bands, an identifiable and prominent feature for this class of materials directly measurable via photoemission [43, 100].

Preprocessing of the 3D band mapping data follows the procedure for $WSe_2$ data described in the main text, except that the rotational symmetrization is only threefold, due to the symmetry of the material. We used numerical initializations from simple functions such as paraboloid and Gaussian in 2D, instead of any first-principles calculation. The reconstructed energy dispersions were smoothed using Chambolle's total variation denoising algorithm [101] implemented in scikit-image [102], removing the high-frequency noise as a result of the Poisson statistics of

the photoemission data. As shown in main text Fig. 6c, the simple initializations we chose are sufficient to reconstruct the complex dispersion from the first two valence bands, the SS and parts of the first conduction band occupied by the excited electronic population. The appearance of the first conduction band for $Bi_2Te_2Se$ is a result of photoexcitation [103]. The reconstructed bands show sixfold symmetry and warping in agreement with previous theoretical investigations [43, 100], which is more straightforwardly visualized in 2D and 3D as in main text Fig. 6d-e. For the dispersion surfaces of the SS and the conduction band, we truncated the dispersion to a realistic energy range not far from the photon energies of the excitation light pulses.

## 3.2 Bulk electronic bands of gold (Au)

The Au dataset was measured at 100 K at the SGM-3 beamline [104] of the 3rd-generation synchrotron radiation facility ASTRID2 in Aarhus, Denmark. The Au samples were purchased from MaTecK GmbH with a (111) surface. The sample preparation procedure has been previously described [105]. The photoemission data were measured along the high-symmetry direction ($\Gamma$KM$\Gamma$) of Au(111), which exhibits a hexagonal symmetry in the surface Brillouin zone [106] (indicated with an overbar over each symmetry label) similar to $WSe_2$. As shown in main text Fig. 6f, the collection of energy bands present in the photoemission data for Au(111) includes the surface states (SSs), which, at sufficient momentum and energy resolution, are composed of momentum-shifted parabolas [94]. The photon energy for the photoemission measurement is $\sim$ 80 eV, which resolves the $sp$ bands and the surface states poorly but the bulk $d$ bands better. The $sp$ bands and the $d$ bands are the low-energy bulk electronic bands of Au.

Before reconstruction, the Au data has been preprocessed using contrast enhancement and intensity smoothing as described in the main text for the $WSe_2$ data before reconstruction. The reconstruction used existing DFT calculations, which feature a Au(111) slab containing five Au layers constructed according to [107], as initialization to retrieve parts of the $d$ bands that are resolvable within the current dataset. The calculation used GPAW code version 1.31 with

GLLBSC functional [108]. The comparison between initialization and reconstruction is shown in main text Fig. 6g. The choice of the initialization is a consistent set of energy bands (i.e. produced by the same slab) from DFT calculations of Au(111) slabs in the energy range close to the noticeable bands in the photoemission data. Although traditionally, slab calculations along with overplotting are used to approximate the total band width, we have shown that our reconstruction approach can detect existing band-like dispersive features in these highly congested data.

### 3.3 Reconstructing the kink anomaly

Kink anomalies are a kind of feature for electron-phonon interaction in photoemission signals [5, 6] found in various materials [97, 98, 109]. To test the reconstruction performance, we simulated the photoemission signal for a kink anomaly using the full spectral function introduced in Eq. (11). The real part of the electron self-energy is calculated using the Eliashberg function [110] represented as an Einstein mode (i.e. single-phonon mode with a delta-function-like frequency response) [111], which appears near the Fermi level. Further details of the computational model can be found in the Jupyter notebooks within the associated compute capsule. The presence of an Einstein mode in the spectral function results in a phonon-induced kink at around - 0.1 eV.

The outcome of the reconstruction, shown in Supplementary Fig. 10, indicates that the MRF model can recover faithfully the quasiparticle dispersion including the shape of the kink anomaly. The reconstruction can simply be initialized with a flat line, which produces identical results from initialization with a linear dispersion that could represent prior knowledge of the algorithm user. The results show that for strongly dispersive energy bands with almost vertical dispersion along the energy direction, reconstruction along the energy direction (i.e. treating the data as a collection of momentum distribution functions) yields a better outcome. This is because the existence of the kink violates the one-to-one mapping between the band energy,

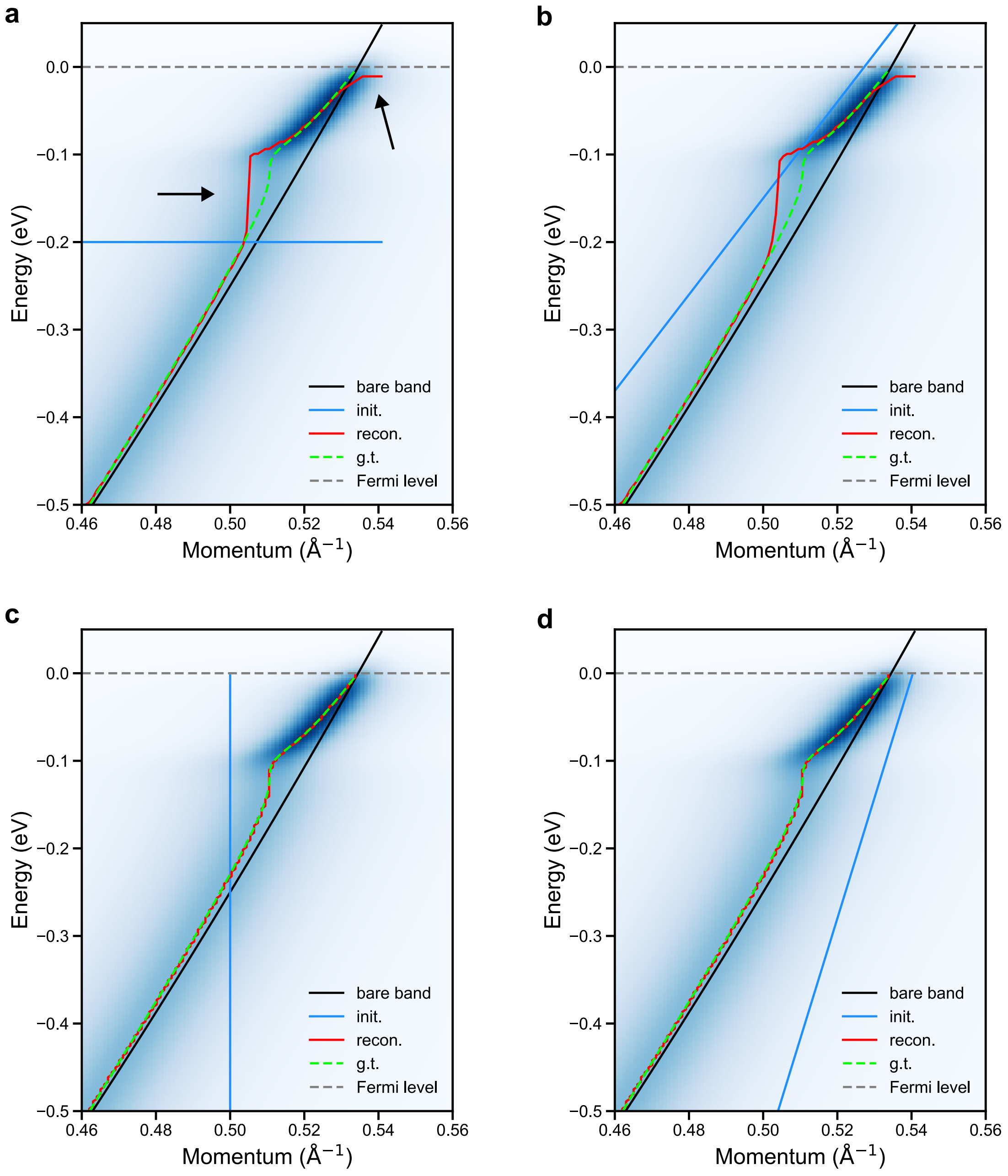


Supplementary Figure 10: **Band reconstruction involving a kink anomaly**. Band reconstruction was carried out along either the momentum (**a**, **b**) or energy (**c**, **d**) directions. Reconstruction (recon.) along the momentum direction using **a**, a flat (uninformative) initialization (init.) and **b**, an informative initialization that approximates foreknowledge of the linear bare-band dispersion yield mostly identical outcomes, which have deviations from the ground truth (g.t.) near the kink and the Fermi level, as indicated with black arrows in **a**. Reconstruction along the energy direction using **c**, a flat (uninformative) initialization and **d**, an informative initialization both yield highly accurate outcomes compared with the ground-truth quasiparticle dispersion in dashed green lines in **a**-**d**.

$E(k)$, and the photoelectron momentum, $k$ (see Supplementary Fig. 10a-b). In these cases, the reconstruction is still viable using the momentum distribution function as the likelihood in the MRF model, which effectively amounts to transposing the image and swapping the momentum and energy coordinates, while the same optimization algorithm described in this work for the EDC-based approach can be reused to obtain the correct quasiparticle dispersion (see Supplementary Fig. 10c-d).

# 4 Band structure calculations

## 4.1 DFT calculations

The crystal structure of bulk $WSe_2$ with $2H$ stacking ($2H$-$WSe_2$) belongs to the $P6_3/mmc$ space group and consists of two Se-W-Se triatomic layers as shown in Supplementary Fig. 11. The stacking order of the two hexagonal layers is -$BAB$-$ABA$- and the long $c$-axis is oriented perpendicular to the layers. Electronic structure calculations were performed within DFT using the local density approximation (LDA), the generalized-gradient approximation (GGA-PBE and GGA-PBEsol), and hybrid (HSE06) exchange-correlation functionals as implemented in FHI-aims [71] (Version 171221_1). The atomic orbitals basis sets, the integration grids and the Hartree potential employed for all calculations are according to the default "tight" numerical settings of FHI-aims. A 16×16×4 uniform **k**-gird was used to sample the Brillouin zone. The Broyden-Fletcher-Goldfarb-Shanno optimization algorithm was used to relax the atomic positions until the residual force component per atom was less than $10^{-2}$ eV/Å. Supplementary Table 3 shows the optimized lattice constants, $a$ and $c$, as obtained by the evaluation of the analytical stress tensor [112] using different exchange-correlation functionals. In all BS calculations, we included the effect of spin-orbit coupling, which is known to introduce a large splitting of the outermost valence states of bulk $2H$-$WSe_2$ [113].

The calculated BSs of bulk $2H$-$WSe_2$ using different levels of approximation for the exchange-

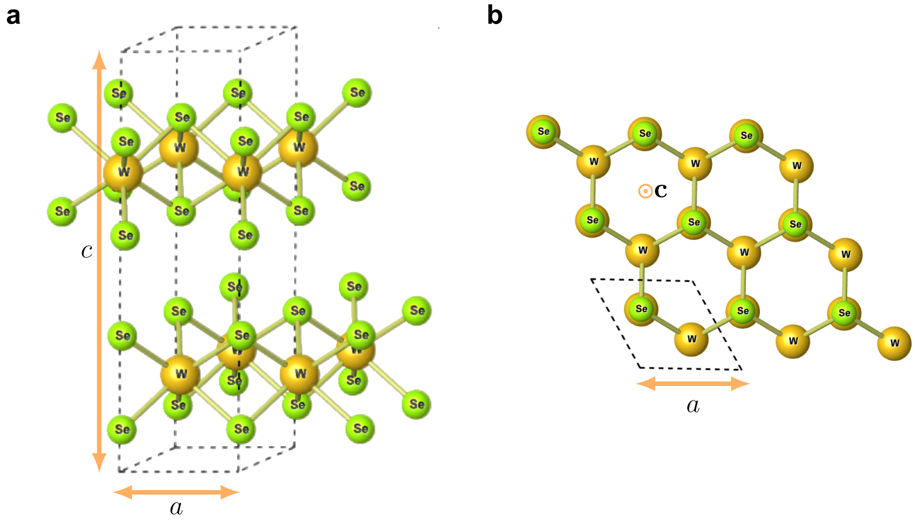


Supplementary Figure 11: **Crystal structure of bulk $2H$-WSe$_2$. a**, Side view and **b**, top view of the crystal structure of $2H$-WSe$_2$. The space group of the hexagonal structure is P6$_3$/mmc with the $c$-axis oriented perpendicular to the stacking layers. In each case, the real-space unit cell is labelled by dashed black lines.

Supplementary Table 3: **Parameters from density functional theory calculations.** Optimized lattice constants, spin-orbit splitting of the topmost valence states at the K high-symmetry point, and the band gap of bulk $2H$-WSe$_2$ calculated within density functional theory using the LDA, PBE, PBEsol and HSE06 exchange-correlation functionals. For comparison, we also report the corresponding experimental values at room temperature.

| xc-functional | LDA | PBE | PBEsol | HSE06 | Experiment |
|---|---|---|---|---|---|
| $a$ (Å) | 3.250 | 3.317 | 3.269 | 3.295 | 3.28 |
| $c$ (Å) | 12.827 | 14.921 | 13.211 | 13.863 | 12.98 |
| Spin-orbit splitting at K (eV) | 0.485 [1]<br>0.490 [2] | 0.473 [1]<br>0.481 [2] | 0.476 [1]<br>0.484 [2] | 0.467 [1]<br>0.480 [2] | 0.5 [3] |
| Band gap (eV) | 1.022 [1]<br>1.052 [2] | 1.186 [1]<br>1.074 [2] | 1.105 [1]<br>1.060 [2] | 1.679 [1]<br>1.582 [2] | 1.219 [4] |

[1] Fully optimized structure.
[2] Optimized structure by fixing the lattice parameters to experimental values.
[3] Ref. [11].
[4] Ref. [114].

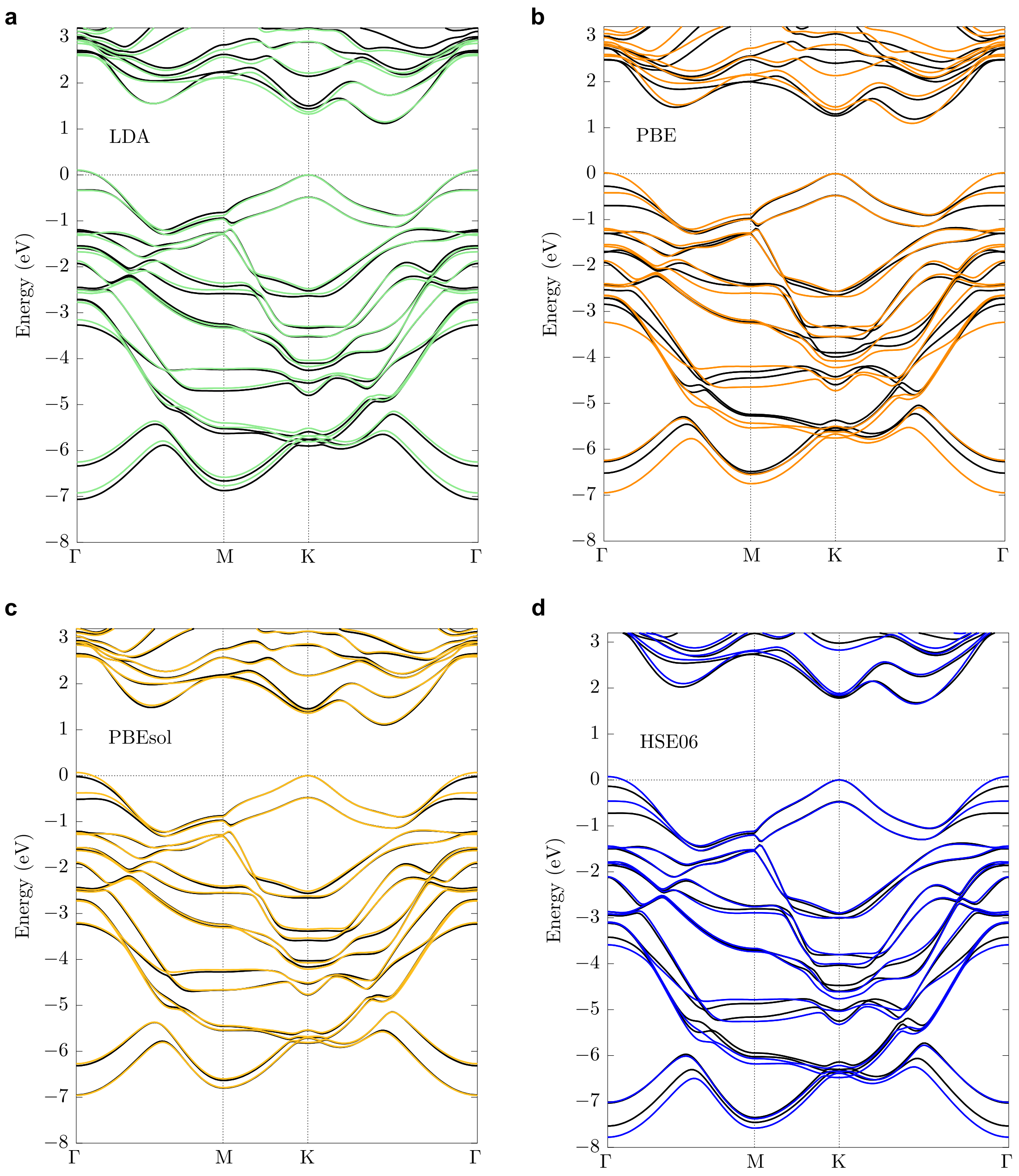


Supplementary Figure 12: **Bulk electronic band structure of 2$H$-WSe$_2$.** **a-d**, Band structure of bulk 2$H$-WSe$_2$ along the Γ-K-M-Γ momentum path of its Brillouin zone including the effect of spin-orbit coupling. Calculations were performed using the LDA (green, **a**), PBE (orange, **b**), PBEsol (yellow, **c**), and HSE06 (blue, **d**) exchange-correlation functionals and optimized structures (see Supplementary Table 3) with the unit cell dimensions kept fixed at the experimental lattice constants. Black lines in **a-d** represent the corresponding calculations using fully optimized geometries. For comparison, the two band structures in each plot are rigidly shifted to align their uppermost valence state at the K high-symmetry point, where we also define as the energy zero. All band structure calculations used $k_z = 0.35$ Å$^{-1}$.

correlation (XC) functional are shown in Supplementary Fig. 12. For each XC functional, the calculations were performed on (1) fully optimized structures (black lines), and on (2) optimized structures by fixing the lattice parameters of the unit cell to the experimental values (colored lines). All calculations using different XC functionals reveal an indirect band gap with the conduction band minimum located along the Γ-K path (Γ and K being the bulk equivalents of the $\overline{\Gamma}$ and $\overline{\mathrm{K}}$ high-symmetry points). For both sets of optimized structures, the LDA results reveal a valence band maximum at the Γ point, compatible with experimental measurements, while the PBE, PBEsol, and HSE06 band structures obtained for fully optimized structures exhibit a valence band maximum at the K point. Nevertheless, fixing the unit cell dimensions at the experimental lattice constants reproduces the experimental behavior that the valence band maximum resides at the Γ point. The difference between the two sets of calculations obtained using PBE, PBEsol, and HSE06 functionals is attributed to the overestimation of the lattice parameter $c$ and the residual strain along the $c$-axis [115]. The calculated indirect band gaps and the spin-orbit splitting of the two topmost valence states at the K point using both sets of optimized structures are shown in Supplementary Table 3.

## 4.2 Brillouin zone tiling

The generation of a large and densely sampled patch of energy bands covering the first Brillouin zone and beyond is crucial for initializing the MRF model. To balance the computational cost using different XC functionals with the dense sampling similar to the experimental data grid, we used the symmetry properties of the Brillouin zone to tile the calculated momentum-space rectangular patch that covers the Γ, K and M points of the Brillouin zone. The hexagonal Brillouin zone of $WSe_2$ has a sixfold rotation symmetry axis and two independent mirror planes in the ($k_x$, $k_y$) coordinates. The initial rectangular patch is first symmetrized about the two mirror planes in the Γ-K and Γ-M directions to form a larger patch, which is then rotated by 60° and 120°, respectively, and combined with the original mirror-symmetrized patch. The

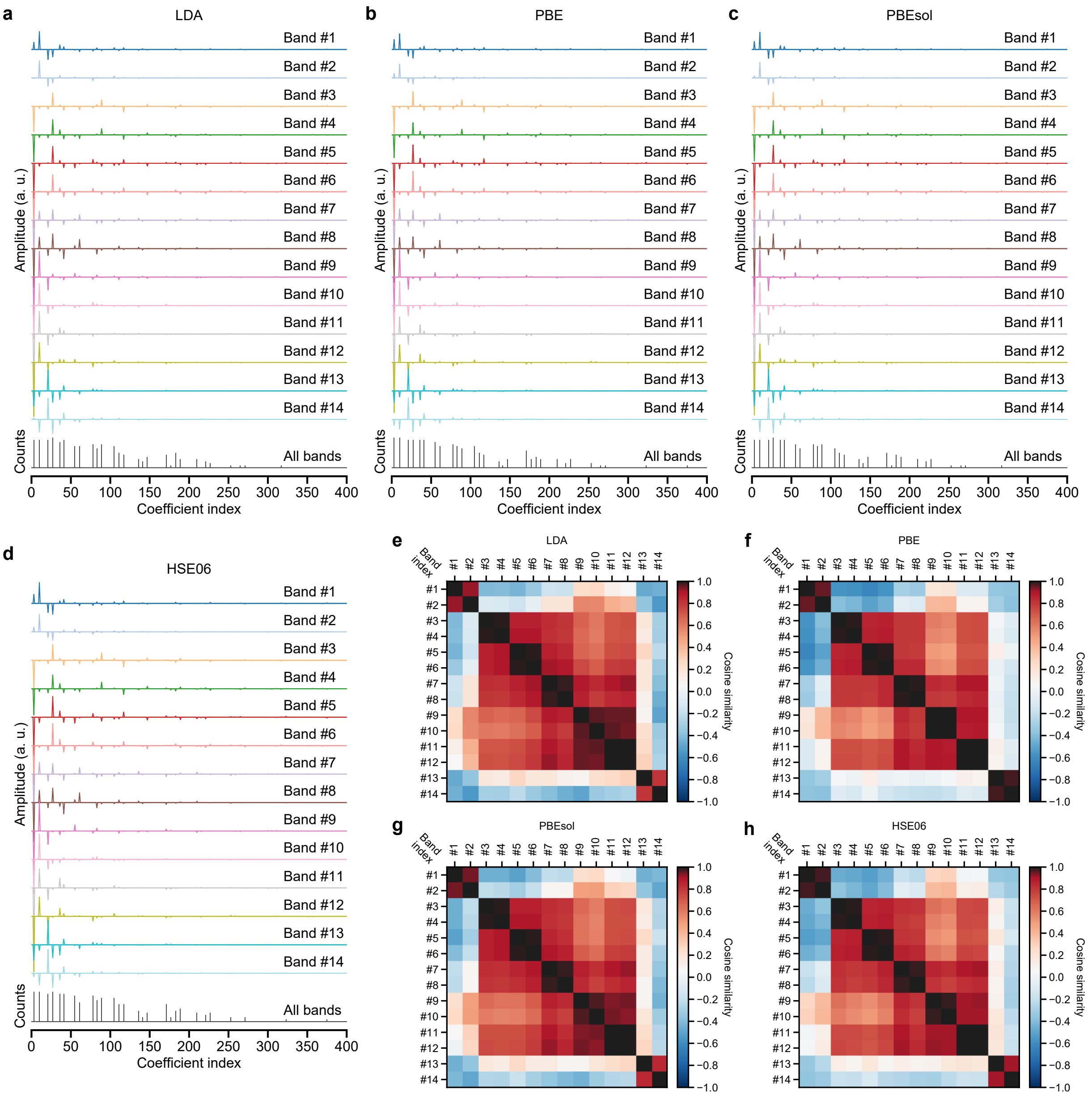


Supplementary Figure 13: **Geometric featurization of the energy bands of $WSe_2$.** **a-d**, Decomposition of the 14 valence energy bands of $WSe_2$ into hexagonal Zernike polynomials for the DFT band structure calculations carried out at the levels of LDA (**a**), PBE (**b**), PBEsol (**c**), and HSE06 (**d**), respectively. Similar characteristics are seen compared with the reconstructed band structure shown in Fig. 3a in the main text, including the sparse distribution of major basis terms and the decreasing dependence on higher-order basis terms. **e-h**, Cosine similarity matrices between the 14 energy bands of $WSe_2$ for the DFT band structure calculations carried out at the levels of LDA (**e**), PBE (**f**), PBEsol (**g**), and HSE06 (**h**), respectively. The characteristics of these matrices resemble that calculated for the reconstructed band structure as shown in Fig. 3c in the main text.

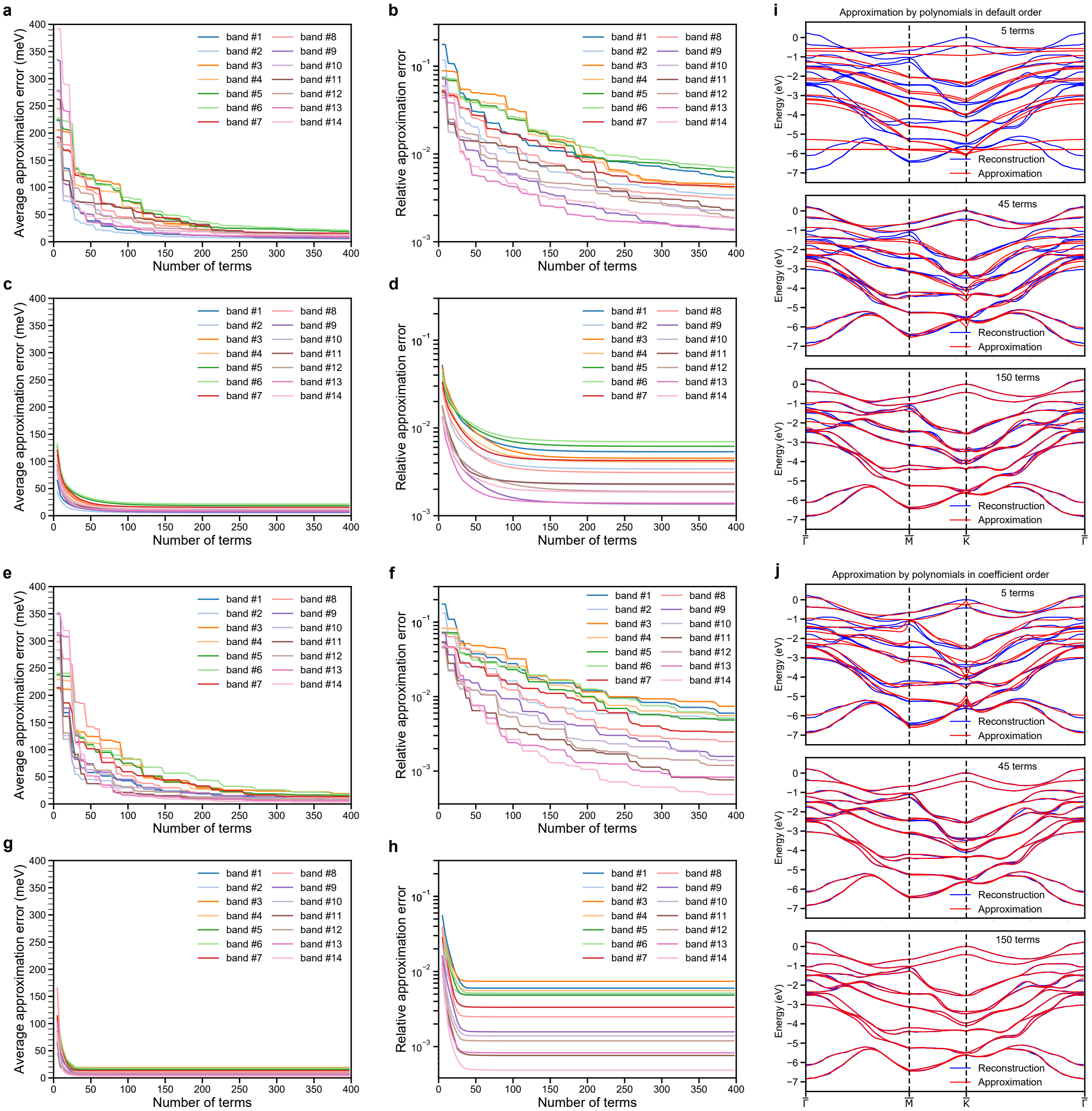


Supplementary Figure 14: **Approximation to the band structure of $WSe_2$ by a polynomial basis**. **a-j**, Demonstration of the convergence properties of the polynomial approximation using reconstructed photoemission band structure (**a-d**) and DFT band structure calculated at the LDA level (**e-h**). When summing the hexagonal Zernike polynomial in the default order, the average and relative approximation errors for the reconstructed (**a**,**b**) and theoretical (**e**,**f**) energy bands converge much slower than summing the polynomials in an ordering ranked by the magnitude of their coefficients (coefficient order). This observation is similar for reconstructed (**c**,**d**) and theoretical (**g**,**h**) energy bands. **i-j**, Visualization of the difference in convergence rates using the reconstructed band structure along the high-symmetry lines. The naturally-ordered polynomial basis has not yet converged with 150 terms (**i**), while the coefficient-ranked polynomials (**j**) produces an accurate approximation well within that limit.

composite patch is then shifted along all six Γ-M directions by one unit cell distance and the result is cut to the required shape compatible with photoemission data.

# 5 Band structure informatics

## 5.1 Global structure descriptors

We use informatics tools for data retrieval, representation and comparison for entire bands. We extend the examples given in main text Fig. 3b to other bands of $WSe_2$ reconstructed in the present work. Supplementary Fig. 13 displays the band-wise comparison of dispersion surfaces within other DFT calculations. These results contain similar features as in main text Fig. 3a and c, reaffirming that the geometric featurization provides a sparse representation of the band dispersions and that the dispersion similarities are largely preserved despite the use of different exchange-correlation functionals in the DFT calculations. They may, therefore, be regarded as general features of the $WSe_2$ band structure.

In Supplementary Fig. 14, we demonstrate numerically the approximation capability of the hexagonal ZP basis set to all 14 valence bands of $WSe_2$. Despite the stark differences in energy dispersion, the approximation to reconstructed bands (Supplementary Fig. 14a-d) and theoretical band structure at the level of LDA-DFT (Supplementary Fig. 14e-h) show comparable convergence rates. Quantitatively, the approximation using hexagonal ZPs ordered by the magnitude of the corresponding coefficients (i.e. coefficient order) converges to within 10-30 meV/band within 50 polynomial basis terms, substantially faster than using the default order (see also Fig. 3b for reference). The remaining errors are on par with the finite step size along the energy axis in the data ($\sim$ 18 meV) that results in the imperfect smoothness of the reconstructed bands. This further proves that the hexagonal ZPs can provide an accurate and sparse approximation for the band structure data. The trend of convergence between these two types of polynomial ordering is further illustrated in Supplementary Fig. 14i-j in the momentum path

along high-symmetry lines of the reconstructed band structure.

## 5.2 Local structure descriptors

Local structural information includes energy gaps, effective masses, warpings, (avoided) crossings, etc. We extracted some of their associated parameters at and around three high-symmetry points ($\overline{\mathrm{K}}$, $\overline{\mathrm{M}'}$, and $\overline{\Gamma}$, see main text Fig. 5a) and compiled the results in Supplementary Table 4. The dispersions and band structure parameters from the MAP reconstruction are compared with those extracted by the line-by-line fitting of the EDCs, which used the band energies from the reconstruction as initialization to improve robustness. Around $\overline{\mathrm{K}}$, two spectral peaks corresponding to two spin-split bands were fit simultaneously, while around $\overline{\mathrm{M}'}$ and $\overline{\Gamma}$, four were fit simultaneously due to the spectral proximity of the first four valence bands (see Supplementary Fig. 5). The fitting is carried out using a linear combination of Voigt lineshapes and the lmfit package [116] with the reconstructed band energy as initialization (but not fixed). The fitting procedure iterates over the EDCs (e.g. a total of $50\times50$ EDCs for the patch around $\overline{\mathrm{M}'}$). Unstable fits yielding erratic results (e.g. if differing greatly from neighboring values) are re-fit with either algorithmically or manually adjusted initialization. Supplementary Table 4 shows that the local structural information from reconstruction is generally consistent with those obtained by iterative pointwise fitting while differing from DFT calculations. The deviations in the size of energy gaps at $\overline{\mathrm{K}}$ and $\overline{\mathrm{M}'}$ between reconstruction and pointwise fitting lie in the same range as the momentum-averaged reconstruction errors (see Supplementary Section 2), which are due to the finite coordinate spacing in the data ($\sim$ 18 meV in energy).

The region extracted around $\overline{\mathrm{K}}$ (see main text Fig. 5d-e) contains about 10% of the distance of $\overline{\Gamma}-\overline{\mathrm{K}}$. Due to the strong trigonal warping (TW) effect in this class of materials, the effective masses and the TW parameters around $\overline{\mathrm{K}}$ were fit simultaneously in 2D using the momentum-

Supplementary Table 4: **Band structure parameters from experiment and theory.** Effective masses of holes ($m_{\overline{\mathrm{K}}}$), trigonal warping parameters ($C$) are extract at $\overline{\mathrm{K}}$ point in the first two valence bands. Two directional effective masses at $\overline{\mathrm{M}'}$ ($m_{\overline{\mathrm{M}'}}$), and one at $\overline{\Gamma}$ ($m_{\overline{\Gamma}}$), are obtained for the first valence band. The energy gaps ($\Delta E$) between the first two valence bands are obtained at both $\overline{\mathrm{K}}$ and $\overline{\mathrm{M}'}$ points. The number (1 or 2) in the subscript of the parameter symbols denotes the valence band index, $m_e$ is the mass of an isolated electron.

| Symmetry point | Parameter | LDA recon. [1] | Line fitting [2] | LDA [3] | HSE06 [3] |
|---|---|---|---|---|---|
| $\overline{\mathrm{K}}$ | $m_{\overline{\mathrm{K}},1}/m_e$ | −0.62 | −0.60 | −0.49 | −0.42 |
| $\overline{\mathrm{K}}$ | $m_{\overline{\mathrm{K}},2}/m_e$ | −0.74 | −0.78 | −0.64 | −0.54 |
| $\overline{\mathrm{K}}$ | $C_{\overline{\mathrm{K}},1}$ (eV·Å$^3$) | 5.3 | 5.8 | 6.2 | 4.5 |
| $\overline{\mathrm{K}}$ | $C_{\overline{\mathrm{K}},2}$ (eV·Å$^3$) | 4.0 | 3.9 | 3.9 | 3.2 |
| $\overline{\mathrm{K}}$ | $\Delta E_{\overline{\mathrm{K}},1-2}$ (meV) | 419 | 446 | 485 | 467 |
| $\overline{\mathrm{M}'}$ | $m_{\overline{\mathrm{M}'}-\overline{\Gamma},1}/m_e$ | 0.71 | 0.72 | 0.25 | 0.17 |
| $\overline{\mathrm{M}'}$ | $m_{\overline{\mathrm{M}'}-\overline{\mathrm{K}'},1}/m_e$ | −1.6 | −1.5 | −1.1 | −0.90 |
| $\overline{\mathrm{M}'}$ | $\Delta E_{\overline{\mathrm{M}'},1-2}$ (meV) | 352 | 338 | 127 | 48 |
| $\overline{\Gamma}$ | $m_{\overline{\Gamma},1}/m_e$ | −0.82 | −1.1 | −0.81 | −1.0 |

[1] Using band dispersion reconstructed globally by the proposed probabilistic machine learning algorithm with DFT calculation at the LDA level as the initialization.
[2] Using band dispersion from iterative lineshape fitting of the energy distribution curves (in a region around the corresponding high-symmetry points).
[3] With fully optimized structure, see Supplementary Table 3.

space model derived from **k**·**p** theory [28].

$$E(\mathbf{q}) = \frac{\hbar^2\mathbf{q}^2}{2m_{\overline{\mathrm{K}}}} + C|\mathbf{q}|^3\cos(3\varphi_{\mathbf{q}} + \theta) + E_0. \tag{25}$$

Here, **q** is the momentum vector **k** recentered on a particular $\overline{\mathrm{K}}$ (or $\overline{\mathrm{K}'}$) point by translation, $m_{\overline{\mathrm{K}}}$ is the effective mass of the hole at $\overline{\mathrm{K}}$ point, $C$ is the magnitude of the TW (named $C_{3\mathrm{w}}$ in [28]), $\varphi_{\mathbf{q}}$ is the polar angle in the coordinate system centered on a $\overline{\mathrm{K}}$ (or $\overline{\mathrm{K}'}$) point, $\theta$ is an auxiliary fitting parameter used to accommodate the orientation of the TW with respect to the pixel coordinates defined by the rectangular region of interest, $E_0$ accounts for the energy offset. The energy gaps at $\overline{\mathrm{K}}$ ($\Delta E_{\overline{\mathrm{K}},1-2}$) and $\overline{\mathrm{M}'}$ ($\Delta E_{\overline{\mathrm{M}'},1-2}$) are illustrated in main text Fig. 5 (b and d), respectively. The $\overline{\mathrm{M}'}$ (or $\overline{\mathrm{M}}$) point situates at a saddle point of the dispersion surface (first

valence band), as shown in main text Fig. 5b-c. Its lower symmetry (compared with $\overline{\mathrm{K}}$, $\overline{\mathrm{K}'}$ and $\overline{\Gamma}$) means that the effective masses exhibits anisotropy, with opposite signs and magnitude along the $\overline{\mathrm{M}'}-\overline{\Gamma}$ and $\overline{\mathrm{M}'}-\overline{\mathrm{K}'}$ directions. We fit the dispersion locally using a model that also accounts for the spin-orbit interaction involving a linear momentum-dependent shift (Eq. 14 in [28]). The second valence band is not fitted at $\overline{\mathrm{M}'}$ due to the pronounced dispersion modulation by interband coupling unaccounted for in the existing saddle-shaped model. At around $\overline{\Gamma}$, a single effective mass is extracted by fitting a paraboloid to a local patch of the dispersion surface.

## References


80. Hüfner, S. *Photoelectron Spectroscopy* 3rd Edition (Springer Berlin Heidelberg, Berlin, Heidelberg, 2003).

81. Bishop, C. M. *Pattern Recognition and Machine Learning* (Springer, 2006).

82. Hammersley, J. M. & Clifford, P. *Markov Fields on Finite Graphs and Lattices* Unpublished. 1971.

83. Besag, J. Spatial Interaction and the Statistical Analysis of Lattice Systems. *Journal of the Royal Statistical Society. Series B (Methodological)* **36,** 192–236 (1974).

84. Geman, S. & Geman, D. Stochastic Relaxation, Gibbs Distributions, and the Bayesian Restoration of Images. *IEEE Transactions on Pattern Analysis and Machine Intelligence* **6,** 721–741 (1984).

85. Besag, J. On the Statistical Analysis of Dirty Pictures. *Journal of the Royal Statistical Society: Series B (Methodological)* **48,** 259–279 (1986).

86. Pearl, J. *Probabilistic Reasoning in Intelligent Systems* (Morgan Kaufmann, 1988).

87. Besag, J. *On the statistical analysis of nearest-neighbours* in *Proceedings of the European Meeting of Statisticians, Budapest* (1972).

88. Locatelli, M. A note on the Griewank test function. *Journal of Global Optimization* **25,** 169–174 (2003).

89. Bena, C. & Montambaux, G. Remarks on the tight-binding model of graphene. *New Journal of Physics* **11,** 095003 (2009).

90. Mahajan, V. N. & Dai, G.-m. Orthonormal polynomials for hexagonal pupils. *Optics Letters* **31,** 2462 (2006).

91. Van de Hulst, H. C. & Reesinck, J. J. M. Line Breadths and Voigt Profiles. *The Astrophysical Journal* **106,** 121 (1947).

92. Zaghloul, M. R. & Ali, A. N. Algorithm 916: Computing the Faddeyeva and Voigt Functions. *ACM Transactions on Mathematical Software* **38,** 1–22 (2011).

93. Dong, S. *et al.* Direct measurement of key exciton properties: Energy, dynamics, and spatial distribution of the wave function. *Natural Sciences* **1,** 1:e10010 (2021).

94. LaShell, S., McDougall, B. A. & Jensen, E. Spin Splitting of an Au(111) Surface State Band Observed with Angle Resolved Photoelectron Spectroscopy. *Physical Review Letters* **77,** 3419–3422 (1996).

95. Xian, R. P. *pesarxiv* `https://github.com/mpes-kit/pesarxiv`.

96. Grohmann, C. H., Smith, M. J. & Riccomini, C. Multiscale Analysis of Topographic Surface Roughness in the Midland Valley, Scotland. *IEEE Transactions on Geoscience and Remote Sensing* **49,** 1200–1213 (2011).

97. Giustino, F., Cohen, M. L. & Louie, S. G. Small phonon contribution to the photoemission kink in the copper oxide superconductors. *Nature* **452,** 975–978 (2008).

98. Verdi, C., Caruso, F. & Giustino, F. Origin of the crossover from polarons to Fermi liquids in transition metal oxides. *Nature Communications* **8,** 15769 (2017).

99. Mi, J.-L. *et al.* Phase Separation and Bulk p-n Transition in Single Crystals of $Bi_2Te_2Se$ Topological Insulator. *Advanced Materials* **25,** 889–893 (2013).

100. Michiardi, M. *et al.* Strongly anisotropic spin-orbit splitting in a two-dimensional electron gas. *Physical Review B* **91,** 035445 (2015).

101. Chambolle, A. An Algorithm for Total Variation Minimization and Applications. *Journal of Mathematical Imaging and Vision* **20,** 89–97 (2004).

102. Van der Walt, S. *et al.* scikit-image: image processing in Python. *PeerJ* **2,** e453 (2014).

103. Papalazarou, E. *et al.* Unraveling the Dirac fermion dynamics of the bulk-insulating topological system Bi2Te2Se. *Physical Review Materials* **2,** 104202 (2018).

104. Hoffmann, S., Søndergaard, C., Schultz, C., Li, Z. & Hofmann, P. An undulator-based spherical grating monochromator beamline for angle-resolved photoemission spectroscopy. *Nuclear Instruments and Methods in Physics Research Section A: Accelerators, Spectrometers, Detectors and Associated Equipment* **523,** 441–453 (2004).

105. Dendzik, M., Bianchi, M., Michiardi, M., Sanders, C. E. & Hofmann, P. Reconstruction-induced trefoil knot Fermi contour of Au(111). *Physical Review B* **94,** 201401 (2016).

106. Tusche, C., Krasyuk, A. & Kirschner, J. Spin resolved bandstructure imaging with a high resolution momentum microscope. *Ultramicroscopy* **159,** 520–529 (2015).

107. Lin, I.-B., Sheu, T. W.-H. & Li, J.-H. Effects of exchange correlation functional on optical permittivity of gold and electromagnetic responses. *Optics Express* **22,** 30725 (2014).

108. Enkovaara, J. *et al.* Electronic structure calculations with GPAW: a real-space implementation of the projector augmented-wave method. *Journal of Physics: Condensed Matter* **22,** 253202 (2010).

109. Ortenzi, L., Cappelluti, E., Benfatto, L. & Pietronero, L. Fermi-Surface Shrinking and Interband Coupling in Iron-Based Pnictides. *Physical Review Letters* **103,** 046404 (2009).

110. Shi, J. *et al.* Direct Extraction of the Eliashberg Function for Electron-Phonon Coupling: A Case Study of Be($10\bar{1}0$). *Physical Review Letters* **92,** 186401 (2004).

111. Allen, P. B. & Dynes, R. C. Transition temperature of strong-coupled superconductors reanalyzed. *Physical Review B* **12,** 905–922 (1975).

112. Knuth, F., Carbogno, C., Atalla, V., Blum, V. & Scheffler, M. All-electron formalism for total energy strain derivatives and stress tensor components for numeric atom-centered orbitals. *Computer Physics Communications* **190,** 33–50 (2015).

113. Voß, D., Krüger, P., Mazur, A. & Pollmann, J. Atomic and electronic structure of $WSe_2$ from *ab initio* theory: Bulk crystal and thin film systems. *Phys. Rev. B* **60,** 14311–14317 (20 1999).

114. Kam, K. K., Chang, C. L. & Lynch, D. W. Fundamental absorption edges and indirect band gaps in $W_{1-x}Mo_xSe_2$ ($0 \leqslant x \leqslant 1$). *Journal of Physics C: Solid State Physics* **17,** 4031–4040 (1984).

115. Desai, S. B. *et al.* Strain-Induced Indirect to Direct Bandgap Transition in Multilayer $WSe_2$. *Nano Letters* **14,** 4592–4597 (2014).

116. Newville, M. *et al.* *lmfit/lmfit-py 1.0.0* https://doi.org/10.5281/zenodo.3588521. 2019.
